# Molecular mechanisms of regulation by a β‐alanine‐responsive Lrp‐type transcription factor from *Acidianus hospitalis*

**DOI:** 10.1002/mbo3.1356

**Published:** 2023-05-22

**Authors:** Amber J. Bernauw, Vincent Crabbe, Fraukje Ryssegem, Ronnie Willaert, Indra Bervoets, Eveline Peeters

**Affiliations:** ^1^ Research Group of Microbiology, Department of Bioengineering Sciences Vrije Universiteit Brussel Brussels Belgium; ^2^ Research Group Structural Biology Brussels, Department of Bioengineering Sciences Vrije Universiteit Brussel Brussels Belgium; ^3^ Alliance Research Group VUB‐UGent NanoMicrobiology, International Joint Research Group VUB‐EFPL NanoBiotechnology & NanoMedicine Vrije Universiteit Brussel Brussels Belgium

**Keywords:** archaea, β‐alanine, leucine‐responsive regulatory protein, protein–DNA interactions, Sulfolobales, transcription regulation

## Abstract

The leucine‐responsive regulatory protein (Lrp) family of transcriptional regulators is widespread among prokaryotes and especially well‐represented in archaea. It harbors members with diverse functional mechanisms and physiological roles, often linked to the regulation of amino acid metabolism. BarR is an Lrp‐type regulator that is conserved in thermoacidophilic Thermoprotei belonging to the order Sulfolobales and is responsive to the non‐proteinogenic amino acid β‐alanine. In this work, we unravel molecular mechanisms of the *Acidianus hospitalis* BarR homolog, Ah‐BarR. Using a heterologous reporter gene system in *Escherichia coli*, we demonstrate that Ah‐BarR is a dual‐function transcription regulator that is capable of repressing transcription of its own gene and activating transcription of an aminotransferase gene, which is divergently transcribed from a common intergenic region. Atomic force microscopy (AFM) visualization reveals a conformation in which the intergenic region appears wrapped around an octameric Ah‐BarR protein. β‐alanine causes small conformational changes without affecting the oligomeric state of the protein, resulting in a relief of regulation while the regulator remains bound to the DNA. This regulatory and ligand response is different from the orthologous regulators in *Sulfolobus acidocaldarius* and *Sulfurisphaera tokodaii*, which is possibly explained by a distinct binding site organization and/or by the presence of an additional C‐terminal tail in Ah‐BarR. By performing site‐directed mutagenesis, this tail is shown to be involved in ligand‐binding response.

## Graphical abstract

Molecular mechanisms of regulation by a beta‐alanine‐responsive Lrp‐type transcription factor from *Acidianus hospitalis*.

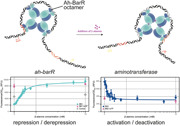

## 1 INTRODUCTION

The Lrp family of transcription factors, named after the prototypical leucine‐responsive regulatory protein (Lrp) from *Escherichia coli*, is a widespread and abundant family of regulators among prokaryotes, especially for the archaea (Peeters & Charlier, 2010; Perez‐Rueda et al., 2018; Yokoyama et al., 2006; Ziegler & Freddolino, 2021). They are also called feast/famine regulatory proteins (FFRPs) (Calvo & Matthews, 1994), referring to the functional role of some family members that regulate the transcription of metabolic genes in response to the availability of nutrients, more specifically amino acids. Other members are involved in the regulation of diverse physiological functions such as transport, antibiotic biosynthesis, motility, DNA repair, and recombination or virulence (Calvo & Matthews, 1994; Chen et al., 2019; Deng et al., 2011; Liu et al., 2017; López‐Torrejón et al., 2006). Lrp‐type regulators have been found to fulfill either a specific or a global role (Friedberg et al., 2001; Kawashima et al., 2008; Unoarumhi et al., 2016), which relates to their intracellular abundance and the regulon size. Some regulators function not only as transcription regulators but also as nucleoid‐associated proteins (NAP), involved in the organization of the chromosome structure (Peterson et al., 2007).

The monomeric structure of an Lrp‐type protein is composed of two domains that are connected by a flexible linker. The N‐terminal DNA‐binding domain (DBD) harbors a helix‐turn‐helix motif or, in most archaeal members, a winged helix‐turn‐helix motif (Peeters & Charlier, 2010). The C‐terminal effector binding domain (EBD), also named the regulation of amino acid metabolism (RAM) domain (Ettema et al., 2002), is characterized by an αβ‐sandwich fold and is of importance for ligand binding and oligomerization of the protein. Archaeal Lrp‐like transcription factors often form higher‐order oligomers, mostly octamers, with the DNA being wrapped around the interacting protein (de los Rios & Perona, 2007; Koike et al., 2004; Kumarevel et al., 2008; Reddy et al., 2007; Thaw et al., 2006). Lrp‐type transcription factors employ different regulatory mechanisms, leading to transcriptional repression and/or activation. Upon interaction with effector molecules, in most cases amino acids, the DNA‐binding affinity and/or transcriptional output is altered via an allosteric response (Kawashima et al., 2008).

In contrast to most Lrp‐type regulators, which are typically responsive to α‐amino acids, a conserved Lrp‐type regulator in the thermoacidophilic Thermoprotei *Sulfolobus acidocaldarius* and *Sulfurisphaera tokodaii* interacts with and responds specifically to the non‐proteinogenic β‐amino acid β‐alanine (Liu et al., 2014). This protein was named BarR, hereafter referred to as Sa‐BarR and St‐BarR for the homologs in *S. acidocaldarius* and *S. tokodaii*, respectively. The BarR‐encoding gene is organized in a conserved divergent operon together with a predicted aminotransferase gene and, in the case of *S. tokodaii*, adjacent to a semialdehyde dehydrogenase gene. In *S. acidocaldarius*, Sa‐BarR was shown to exert a β‐alanine‐dependent transcriptional activation of the divergently oriented aminotransferase gene and a β‐alanine‐independent transcriptional autoactivation of its gene (Liu et al., 2014). This was corroborated by the observation that Sa‐BarR interacts with the *sa‐barR*‐aminotransferase intergenic region in vivo, irrespective of the presence of β‐alanine in the growth medium (Liu et al., 2016). On the contrary, the addition of β‐alanine caused dissociation of the Sa‐BarR‐DNA complex in in vitro experiments (Liu et al., 2014). Sa‐BarR and St‐BarR form octameric structures and the *barR*‐aminotransferase intergenic region harbors multiple binding sites for the regulator, characterized by a 15‐base‐pair (bp) semipalindromic binding motif. It is hypothesized that each binding site is contacted by a dimeric portion of the protein and that the DNA is wrapped around a BarR octamer during interaction (Liu et al., 2014).

BarR displays a high sequence identity with Grp, a glutamine‐responsive Lrp‐type regulator in *S. tokodaii* (Kumarevel et al., 2008). Grp harbors 69% amino acid sequence identity with St‐BarR and Sa‐BarR and it is therefore assumed that Grp and St‐BarR are the result of a gene duplication event in *S. tokodaii* (Kumarevel et al., 2008). The crystal structure of Grp has been used as a template in the structural modeling of Sa‐BarR (Liu et al., 2014). BarR is conserved in other Sulfolobales, including *Acidianus hospitalis*, a species that also has an acidothermophilic lifestyle, growing optimally at temperatures between 65°C and 95°C and a pH between 2 and 4 (You et al., 2011). *A. hospitalis* has a facultative anaerobic chemolithoautotrophic metabolism that relies on sulfur. As compared to model species belonging to Sulfolobales, *A. hospitalis* has not been extensively investigated: only a single protein (Aho7c) has been characterized in detail thus far (Kalichuk et al., 2017).

In this work, the *A. hospitalis* BarR homolog, referred to as Ah‐BarR, is investigated. The DNA wrapping hypothesis is further examined by performing high‐resolution contact probing of the interaction between Ah‐BarR and its operator DNA and an AFM visualization of the conformation of the formed nucleoprotein complexes. Moreover, transcription regulatory mechanisms are studied in‐depth by using a reporter gene system in a bacterial host, and by employing a mutagenesis approach, structural determinants of β‐alanine ligand response are identified in Ah‐BarR.

## 2 MATERIALS AND METHODS

### 2.1 Bioinformatic analyses and structural predictions

Clustal Omega (Madeira et al., 2022; Sievers et al., 2011) was used for protein sequence alignment, and MUSCLE (Edgar, 2004; Madeira et al., 2022) for DNA sequence alignment of intergenic promoter regions. Protein structures were predicted with AlphaFold (Jumper et al., 2021) and homology modeling was done with SWISS‐MODEL (Waterhouse et al., 2018). Visualizations of protein structures, as well as structural alignments, were performed in PyMOL (Schrödinger & Delano, 2020). Transcription factor binding sites were predicted using FIMO (Grant et al., 2011). Sequence logos were created with WebLogo 3 (Crooks et al., 2004), using both the forward and reverse sequences. Molecular docking of ligand interaction was done with the program AutoDock Vina (Trott & Olson, 2010).

### 2.2 Cloning and site‐directed mutagenesis

**Figure A1:**
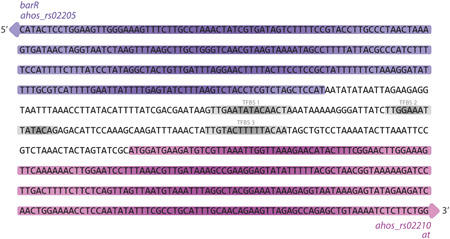
DNA sequence of gb_Ahos. This 750 bp sequence is part of the *Acidianus hospitalis* genome and contains (parts of) the genes *ahos_rs02205 (ah‐barR)* and *ahos_rs02210* (aminotransferase gene) and the intergenic region. Predicted binding sites are indicated in gray.

The *ah‐barR* coding sequence (*ahos_rs02205*) was codon‐optimized for *E. coli* and a construct was designed in which it was fused to linkers containing homology regions to the insertion site on pET24a. This construct was ordered at Twist Bioscience, as well as a synthetic DNA fragment of gb_Ahos (Figure A1). Genomic DNA of *S. acidocaldarius* was used as a template for amplification of the *saci_rs10330‐saci_rs10335* region. A detailed overview of the used primers, restriction enzymes, and constructs generated in this work is provided in Tables A1, A2, A3.

Cloning of the *barR* gene, gb_Ahos, or promoters in the different plasmid vectors was performed as described (Bernauw et al., 2022). PCRs were performed using KAPA HiFi DNA polymerase (Roche), plasmids were restricted using FastDigest restriction enzymes (Thermo Scientific) and all reactions were analyzed by agarose gel electrophoresis and purified using the Wizard® SV Gel and PCR Clean‐Up System (Promega). SLiCE (Seamless Ligation Cloning Extract) method (Zhang et al., 2012) was performed as described (Bernauw et al., 2022), followed by heat shock transformation in competent *E. coli* DH5α or MG1655 cells. Colony PCR and Sanger sequencing (Eurofins Genomics) were employed to verify the sequences of the constructs.

pACYC184 gb_Ahos with mutated promoter sequences (mut1, mut2, and mut3), as well as pET24a and pITC plasmids containing mutated Ah‐BarR sequences (M103A, M103N, M103T, T134A, and T136A) were generated according to the site‐directed mutagenesis method described by Edelheit et al. (2009). To this end, PCRs were performed using 500 ng of the restricted wild type (WT) plasmid, 40 pmol of a single primer (either forward or reverse), and KAPA HiFi DNA polymerase (Roche). Corresponding forward and reverse reaction products were combined, followed by a denaturation and annealing step, digestion by the addition of 30 units FastDigest DpnI restriction enzyme (Thermo Scientific), and incubation for 2 h at 37°C. The truncated Ah‐BarR mutants were generated by first amplifying the truncated gene using PCR and by restriction with FastDigest restriction enzymes (Thermo Scientific) of the plasmid backbone, followed by Gibson Assembly cloning using NEBuilder® HiFi DNA Assembly Master Mix (New England Biolabs Inc.). Finally, all mixtures were transformed by heat shock transformation in *E. coli* DH5α chemically competent cells.

### 2.3 Protein expression, purification, and size exclusion chromatography

To perform heterologous expression of Ah‐BarR WT and mutant proteins, the pET24a *ah‐barR* (WT/mutant) plasmids were first transformed in chemically competent cells of *E. coli* SoluBL21. A 300 mL culture was grown at 37°C to an optical density (OD_600nm_) of 0.6, after which the cells were induced with 1 mM of isopropyl β‐D‐1‐thiogalactopyranoside (IPTG) and further incubated for 16 h at 37°C. Cells were pelleted and resuspended in buffer A (100 mM Tris‐HCl pH 8.0, 500 mM NaCl, 40 mM imidazole), followed by the addition of 4 mM Pefabloc® (Roche) and sonication using a Vibracell 75043 (Bioblock Scientific) at 4°C and 20% of the maximal amplitude during 15 min. Next, lysed cells were centrifuged, and the supernatant was subjected to an additional heat treatment for 10 min at 75°C.

After centrifugation, the remaining supernatant was used for purification of the C‐terminally His‐tagged Ah‐BarR proteins by affinity chromatography using a 1 mL HisTrap FF column (Cytiva), coupled to an ÄKTA FPLC system (Cytiva) equipped with a UPC‐900 monitor (Cytiva). Equilibration of the column was done with buffer A, while a linear gradient of 0%–100% buffer B (100 mM Tris‐HCl pH 8.0, 500 mM NaCl, 500 mM imidazole) was applied over 40 column volumes to elute the His‐tagged protein. Eluted fractions were analyzed by sodium dodecylsulfate–polyacrylamide gel electrophoresis (SDS‐PAGE) and the Ah‐BarR‐containing fractions were dialyzed in storage buffer (20 mM Tris‐HCl pH 8.0, 200 mM NaCl). All proteins were concentrated using Vivaspin® 2 (MWCO 5000, Sartorius) up to a concentration between 0.7 and 1 mg/mL.

SEC‐MALS analysis was performed on 30 µL of each protein preparation on a Superdex 200 increase 5/150 GL column (Cytiva), coupled to an HPLC Alliance system (Waters) equipped with a 2998 PDA detector (Waters), a TREOS II MALS detector (Wyatt Technology) and a RI‐501 refractive index detector (Shodex). Additionally, 0.7 mg of WT BarR protein was analyzed in the absence and presence of 100 mM β‐alanine in an SEC experiment using a HiLoad® 16/60 Superdex®200 prep grade column (Cytiva), coupled to an ÄKTA FPLC system (Cytiva) equipped with a UPC‐900 monitor (Cytiva).

### 2.4 Electrophoretic mobility shift and footprinting assays

Electrophoretic mobility shift assays (EMSAs) were performed as described (Charlier & Bervoets, 2022). Either the forward or the reverse primer was radioactively labeled, using fresh γ‐^32^P‐ATP (Perkin Elmer) and T4 polynucleotide kinase (Thermo Scientific). Labeled promoter fragments were obtained by PCR using Taq DNA polymerase (Promega), a ^32^P‐labeled primer, a non‐labeled second primer, and a plasmid template (Table A4). Labeled fragments were purified by acrylamide gel electrophoresis. EMSA reactions were prepared in protein‐DNA binding buffer (20 mM Tris‐HCl pH 8.0, 50 mM NaCl, 0.4 mM EDTA, 0.1 mM DTT, 1 mM MgCl_2_, 12.5% glycerol) with each reaction containing 1 µL (20 cps/µL) of ^32^P‐labeled promoter fragment, an excess (25 µg/mL) of non‐labeled, nonspecific competitor DNA (sonicated salmon sperm DNA, Invitrogen), and purified Ah‐BarR protein. Amino acids were added to the reactions in the mentioned concentrations. Reactions were incubated for 25 min at 37°C, before gel electrophoresis using a 6% native polyacrylamide gel. Gels were visualized using a Storage Phosphor Screen BAS‐IP MS (Cytiva) and Personal Molecular Imager (PMI) system (Bio‐Rad). Scans of the gels were analyzed by densitometry using ImageJ (Schneider et al., 2012), after which GraphPad PRISM (version 9.3.1 for Windows, GraphPad Software, www.graphpad.com) was used to perform Hill curve fitting using nonlinear regression for saturated binding (one site, specific binding), and to determine apparent equilibrium dissociation constants *K*_D_.

“In‐gel” Cu‐OP footprinting was performed as described (Charlier & Bervoets, 2022). First, an EMSA experiment was performed with each reaction containing 300 cps of ^32^P‐labeled promoter fragment (bottom strand labeled) (Table A4) and an Ah‐BarR octameric protein concentration of 0, 108, and 216 nM. After electrophoresis, the gel was immersed in 200 mL of 10 mM Tris‐HCl, pH 8.0, followed by the addition of a 20 mL solution, composed of 1 mL 40 mM 1,10‐phenanthroline (in ethanol), 1 mL 9 mM CuSO_4_ and 18 mL of nuclease‐free water. After 5 min, 10 mL of 100X diluted 3‐mercaptopropionic acid was added and 10 min later, 20 mL of a 30 mM neocuproine solution was added. After 5 min of incubation, the gel was rinsed, an X‐ray‐sensitive film was exposed to the gel for 2 h and the bands corresponding to input DNA and the Ah‐BarR‐DNA complexes were recovered from the gel. A, T, G, and C ladders were prepared using the USB® Thermo Sequenase Cycle Sequencing kit (Applied Biosystems). All samples were loaded on a 6% denaturing polyacrylamide gel and electrophoresis was performed. Gels were visualized using a Storage Phosphor Screen BAS‐IP MS (Cytiva) and Personal Molecular Imager (PMI) system (Bio‐Rad). Densitometry of the footprint pattern was performed using ImageJ (Schneider et al., 2012), after which the ratio of unbound to bound DNA was calculated for each of the individual bands.

### 2.5 AFM

Before AFM, a PCR was performed using KAPA HiFi DNA polymerase (Roche Diagnostics) to generate the 780 bp operator fragment (Table A4), followed by a purification using the Wizard® SV Gel and PCR Clean‐Up System (Promega). Purified DNA and Ah‐BarR protein were diluted in protein‐DNA binding buffer to concentrations of respectively 25 nM DNA and 49 nM Ah‐BarR (octameric concentration). Equal volumes of DNA and protein were mixed and incubated for 15 min at 37°C, after which 2 µL of the reaction was mixed with 28 µL of adsorption buffer (40 mM HEPES pH 7.1, 10 mM NiCl_2_). Twenty microliters of this mixture were deposited on a freshly cleaved mica sheet, followed by 10 min of incubation. Subsequently, the mica surface was extensively rinsed with washing buffer (20 mM HEPES pH 7.4, 3 mM NiCl_2_), after which 200 µL of washing buffer was added. Next, visualization was performed in liquid using an AFM microscope (NanoWizard 4 Ultraspeed 2, Bruker‐JPK). Images were acquired using FASTSCAN‐D probes (resonance frequencies 80–140 kHz and nominal spring constant of 0.25 N/m, Bruker) in AC Mode Fast Imaging. Scan sizes ranged between 170 × 170 nm and 2 × 2 µm. Images were processed through the JPK Data Processing software and Gwyddion (leveling data, correcting scars, and adapting the color scheme). All images were obtained using the same sample and probe. Images of all complexes are available in the Appendix.

### 2.6 Reporter gene assays

Reporter gene assays were performed as previously described (Bernauw et al., 2022). pPRC6 and pITC variants (Table 1) were co‐transformed in *E. coli* MG1655 chemical competent cells using heat shock transformation. Transformant colonies of the Ah‐BarR biosensor strains were picked to inoculate wells of a transparent 96‐well plate (Greiner Bio‐One), each well filled with 200 µL of MOPS EZ Rich Defined medium (Teknova), prepared without the addition of the 5X EZ Supplement, after which the plate was incubated overnight in a ThermoMixer C (Eppendorf) at 30°C and 300 rpm. Next, a black 96‐well plate was filled with a naringenin solution prepared in ethanol (250 mg/L or 2.5 g/L), after which a Breathe‐Easy sealing membrane (Sigma‐Aldrich) was applied and the ethanol was evaporated in a ThermoMixer C (Eppendorf) at 75°C. Twenty microliters of an amino acid solution (β‐alanine or other) were added to the wells in the mentioned final concentrations, as well as 170 µL of MOPS EZ Rich Defined medium (Teknova), prepared without the addition of the 5X EZ Supplement, and 10 µL of a 15X diluted preculture. Finally, the plate was sealed again using a Breathe‐Easy sealing membrane (Sigma‐Aldrich).

**Table 1:** Overview of the used biosensor strains in this work.

Name	pITC plasmid	pPRC6 plasmid
BS1	pITC *ah‐barR*	pPRC6 P_*ah‐barR*_
BS1 noTF	pITC	pPRC6 P_*ah‐barR*_
BS1 M103A	pITC *ah‐barR* M103A	pPRC6 P_*ah‐barR*_
BS1 M103N	pITC *ah‐barR* M103N	pPRC6 P_*ah‐barR*_
BS1 M103T	pITC *ah‐barR* M103T	pPRC6 P_*ah‐barR*_
BS1 T134A	pITC *ah‐barR* T134A	pPRC6 P_*ah‐barR*_
BS1 T136A	pITC *ah‐barR* T136A	pPRC6 P _*ah‐barR*_
BS1 trunc	pITC *ah‐barR* trunc	pPRC6 P_*ah‐barR*_
BS2	pITC *ah‐barR*	pPRC6 P_*ah‐at*_
BS2 noTF	pITC	pPRC6 P_*ah‐at*_
BS3	pITC *sa‐barR*	pPRC6 P_*sa‐barR*_
BS3 noTF	pITC	pPRC6 P_*sa‐barR*_
BS4	pITC *sa‐barR*	pPRC6 P_*sa‐at*_
BS4 noTF	pITC	pPRC6 P_*sa‐at*_
Control	pITC	pPRC6

Plates were incubated at 30°C and 300 rpm for 40 h in a Synergy H1 microplate reader (BioTek) with measurements of OD_600_ and fluorescence (excitation wavelength: 588 nm, emission wavelength: 633 nm) being taken every 20 min. The gain was set to 140 for all measurements, except for the measurements with the amino acids (pools and separately), where the gain was set to 150. For each combination of biosensor strain and β‐alanine concentration, four biological replicates were analyzed. In addition, wells solely containing medium were measured as well to correct for the background signal.

Reporter expression was analyzed at t = 30 h, when all cultures had reached the stationary phase, by taking into account three subsequent time points (*t*–1, *t*, *t*+1). The corrected FL/OD_600_ value was calculated for each replicate (*n* = 4) as follows:

$${\left(\frac{\mathrm{FL}}{OD}\right)}_{\mathrm{cor}}=\frac{\frac{{\mathrm{FL}}_{t-1}-{\mathrm{FL}}_{\mathrm{med},t-1}}{OD_{t-1}-OD_{\mathrm{med},t-1}}+\frac{{\mathrm{FL}}_{t}-{\mathrm{FL}}_{\mathrm{med},t}}{OD_{t}-OD_{\mathrm{med},t}}+\frac{{\mathrm{FL}}_{t+1}-{\mathrm{FL}}_{\mathrm{med},t+1}}{OD_{t+1}-OD_{\mathrm{med},t+1}}}{3}$$

Response curves were fitted to a Hill function of the shape

$$\;P\left(M\right)=\left\{\begin{array}{l} b+a\frac{M^{n}}{\theta^{n}+M^{n}}\;\text{for}\;a\;\beta -\text{alanine}-\text{inducible response}\;\\ b+a\frac{\theta^{n}}{\theta^{n}+M^{n}}\;\text{for}\;a\;\beta -\text{alanine}-\text{repressible response} \end{array}\right.$$

according to a previously published procedure (Landry et al., 2018) with minor adaptations. In this equation, *P* represents the normalized fluorescent output $\frac{\mathrm{FL}}{\mathrm{OD}}$ as a function of *M*, the β‐alanine concentration, b refers to the basal output, and a to the maximum increase in output. The threshold θ represents the β‐alanine concentration for which 50% of the maximum output is attained, relative to the basal level and n is the Hill coefficient. Briefly, lmfit 1.1.0 was used to fit all replicates of a response curve to the appropriate Hill function using the Levenberg‐Marquardt algorithm (Moré, 1978; Newville et al., 2016). Residuals were weighted by multiplying each residual by the inverse of the mean normalized fluorescence ${\left(\frac{\overset{-}{F}L}{OD}\right)}^{-1}$ at the corresponding β‐alanine concentration to fit low and high $\frac{\mathrm{FL}}{\mathrm{OD}}$ values equally well. The results of fitting were displayed by plotting the experimental data along with the fitted Hill function using Matplotlib 3.6.2 on a symmetrical logarithmic scale with the linthresh parameter set to 2, which draws up the x‐axis with a linear area (from 0 to 2 mM) and a logarithmic area (from 2 to 10 mM) (Hunter, 2007). All best‐fit parameters are supplied in Table 2 along with their 95% confidence intervals calculated using the conf_interval function, which performs an *F*‐test. The dynamic range was estimated as $\mu =\frac{a}{b}$.

**Table 2:** Dose‐response parameters for Ah‐BarR biosensor strains.

Biosensor	Basal fluorescence output *b* (RFU)	Maximum fluorescence increase *a* (RFU)	Dynamic range µ	Threshold θ (mM)	Hill coefficient *n*
BS1	2226	6828	3.07	0.37	1.77
	(2054–2389)	(6242–7456)		(0.32–0.42)	(1.51–2.09)
BS1 M103A	2289	7429	3.25	1.35	1.88
	(2161–2406)	(6569–8493)		(1.11–1.77)	(1.50–2.32)
BS1 M103N	2281	9373	4.11	0.46	1.48
	(1922–2616)	(7860–11,176)		(0.36–0.64)	(1.13–1.98)
BS1 M103T	3184	7915	2.49	0.32	1.36
	(2874–3486)	(7039–8890)		(0.26–0.39)	(1.11–1.67)
BS1 T134A	2145	8703	4.06	1.61	1.68
	(2021–2257)	(7668–10,138)		(1.30–2.22)	(1.36–2.04)
BS1 T136A	2469	8171	3.31	0.96	1.36
	(2278–2644)	(7169–9476)		(0.76–1.32)	(1.09–1.67)
BS1 trunc	2485	8840	3.56	0.26	1.60
	(2119–2838)	(7609–10,210)		(0.20–0.33)	(1.27–2.06)
BS2	313	494	1.58	0.11	1.62
	(277–343)	(369–629)		(0.07–0.16)	(1.08–2.52)
BS3	4595	5355	1.17	0.10	2.00
	(4050–5134)	(4567–6254)		(0.08–0.13)	(1.27–3.26)
BS4	13,873	71,426	5.15	0.09	2.99
	(12,733–14,938)	(57,150–87,419)		(0.07–0.11)	(2.33–4.05)

*Note*: Reported values are best‐fit values for fits to a Hill function, together with the 95% confidence interval indicated between brackets (see also Section 2.6).

Statistical analysis of the FL/OD_600_ values was performed in R, using the packages ggplot2, ggpubr, tidyverse, broom, and AICcmodavg. One‐way analysis of variance [ANOVA] was performed, testing the significance of differences between all conditions measured for one biosensor. After that, a Tukey's HSD (honestly significant difference) test was performed to assess the significance between specific conditions.

## 3 RESULTS

### 3.1 Ah‐BarR is an octameric protein with typical Lrp‐type structural features

**Figure 1:**
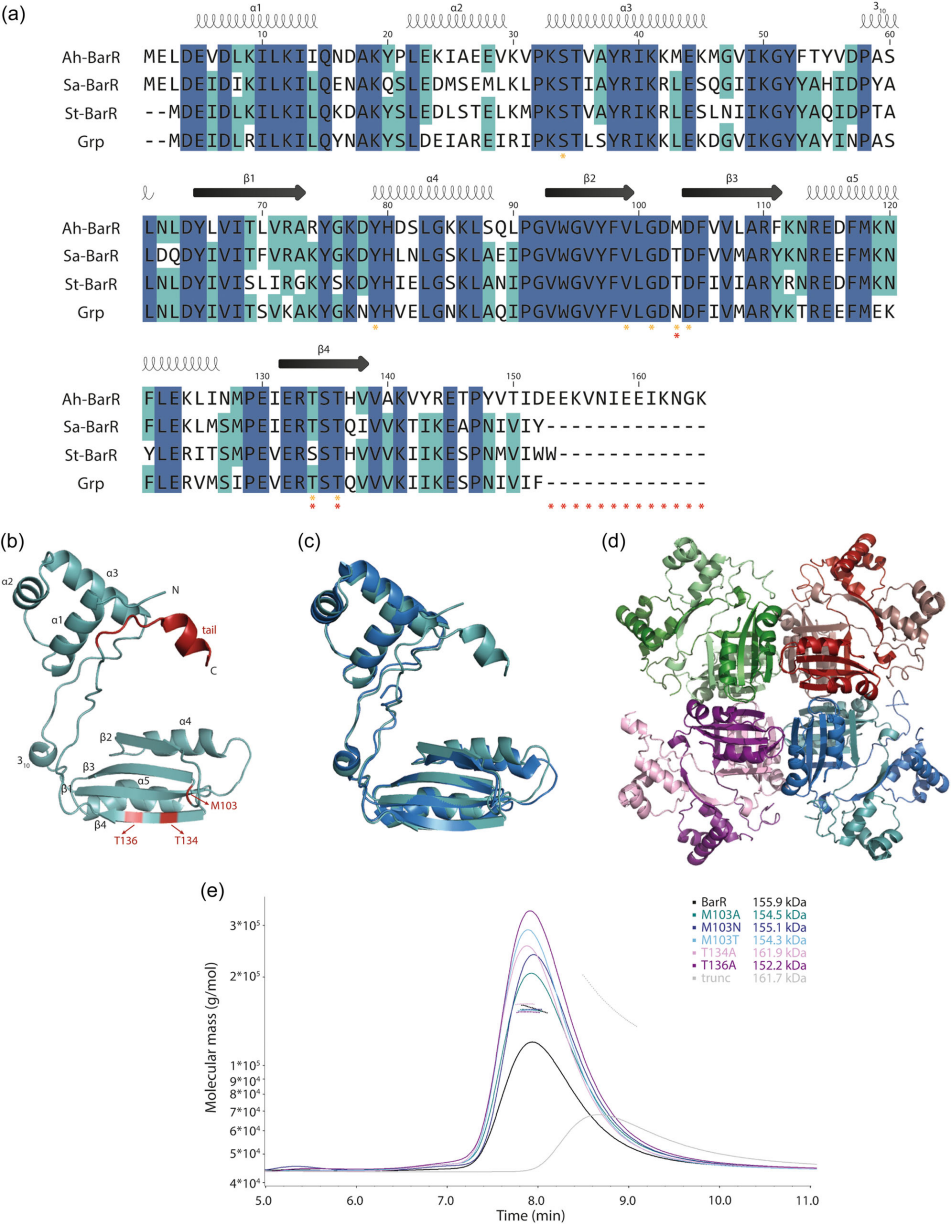
Ah‐BarR is a typical leucine‐responsive regulatory protein (Lrp)‐type transcription factor that adopts an octameric oligomeric state in solution. (a) Amino acid sequence alignment of Ah‐BarR and its homologs Sa‐BarR, St‐BarR, and Grp. Conserved residues are marked in blue, and a prediction of secondary structure elements is shown above the sequences. Residues predicted to be involved in ligand binding are marked with a yellow asterisk, while residues mutated in this work are marked with a red asterisk. (b) Monomeric structure of Ah‐BarR, with α helices, β strands, and N and C termini labeled. Residues mutated in this work are colored red. (c) Alignment of the monomeric structures of Grp (PDB: 2E7W) (Kumarevel et al., 2008) and Ah‐BarR, colored dark and light blue, respectively. (d) Structural model of an Ah‐BarR octamer. Monomeric subunits are colored differently. (e) Size exclusion chromatography‐multi‐angle light scattering (SEC‐MALS) of Ah‐BarR wild‐type and mutant proteins to determine the oligomeric state in solution. The chromatogram displays the light scattering (LS) curves, together with the corresponding molecular weight.

Ah‐BarR (AHOS_RS02205) displays amino acid sequence identities of 66%, 63%, and 64% with its homologs Sa‐BarR from *S. acidocaldarius*, St‐BarR from *S. tokodaii* and Grp from *S. tokodaii*, respectively (Figure 1a). Upon predicting its monomeric structure in AlphaFold, it was confirmed that Ah‐BarR harbors typical structural characteristics of an Lrp‐type protein (Figure 1b). The N‐terminal DBD folds into a helix‐turn‐helix motif, composed of helices α1‐α3, while the C‐terminal EBD harbors the typical αβ‐sandwich fold (β1, α4, β2, β3, α5, and β4). In comparison to Sa‐BarR, St‐BarR, and Grp, Ah‐BarR has an additional C‐terminal tail of 13 amino acids (Figure 1a,b). For the remainder of the protein structure, Ah‐BarR is predicted to display a high degree of structural similarity with its homologs. Indeed, the Ah‐BarR model can be structurally aligned with the Grp crystal structure with an RMSD of 0.751 Å (Figure 1c). Based on the octameric conformation of *S. tokodaii* St‐BarR and Grp (Kumarevel et al., 2008; Liu et al., 2014), the Ah‐BarR structure was also modeled as an octamer (Figure 1d). To experimentally investigate the oligomeric state of Ah‐BarR, size exclusion chromatography was performed in combination with multi‐angle light scattering (SEC‐MALS) (Figure 1e). This revealed a homogenous population with a predicted molecular weight (MW) of 155.9 kDa, confirming an octameric state.

### 3.2 Ah‐BarR interacts with three distinct binding sites in the *barR*‐aminotransferase intergenic region

Sa‐BarR and St‐BarR were shown to interact with multiple binding sites in the intergenic region of the divergent operon of the *barR* gene itself and a putative aspartate aminotransferase gene (Liu et al., 2014). These binding site sequences were used as an input to search for putative Ah‐BarR binding sites in the corresponding genomic region of *A. hospitalis*. This led to the prediction of three regularly spaced 15‐base pair (bp) sites with a semipalindromic nature: 5′‐TTGAATATACAACTA‐3′ (transcription factor binding site (TFBS) 1), 5′‐TTGGAAATTATACAG‐3′ (TFBS 2) and 5′‐TTGTACTTTTTACAA‐3′ (TFBS 3) (Figure A1).

**Figure 2:**
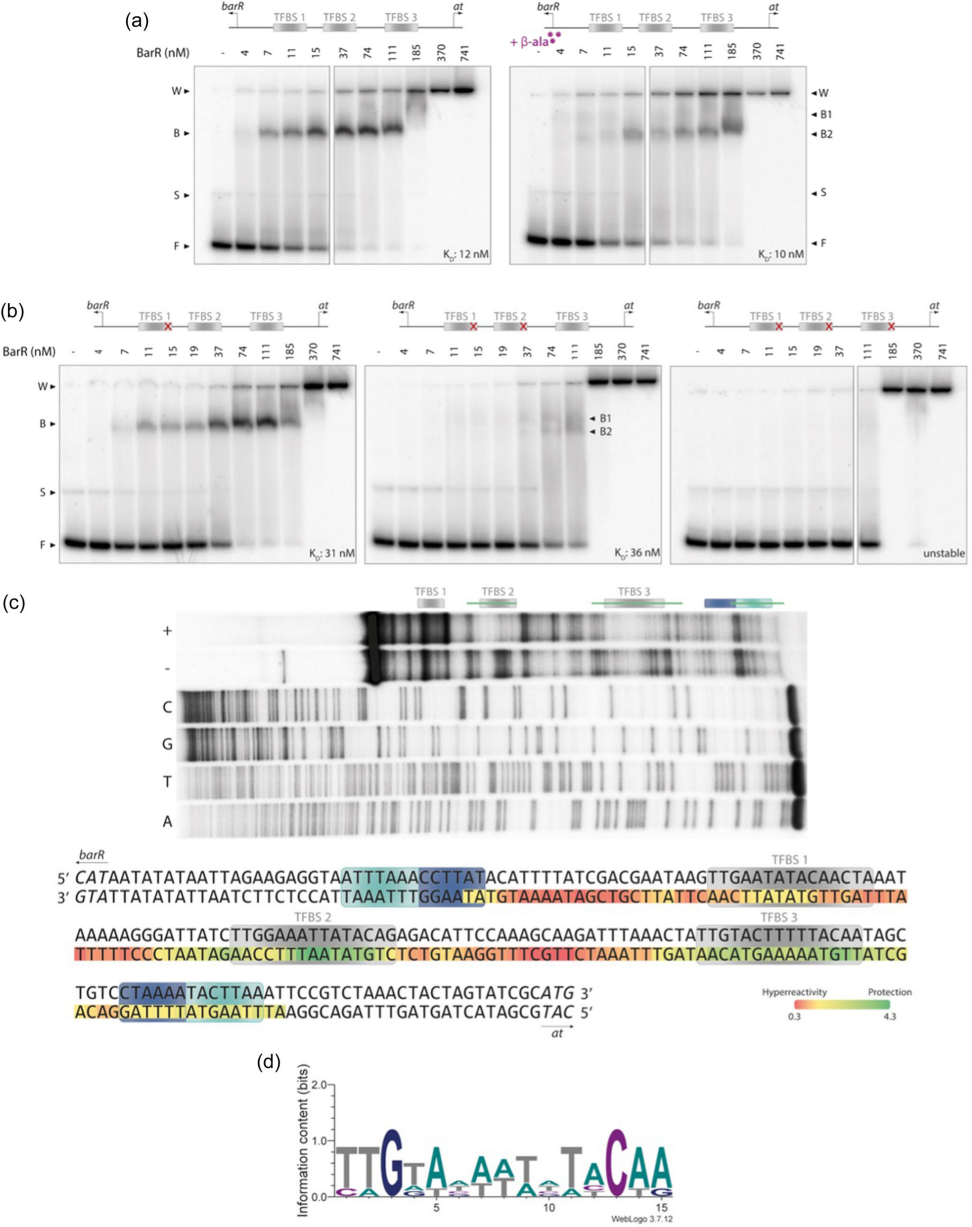
In vitro analysis of Ah‐BarR binding to the barR‐aminotransferase intergenic region. TFBS, transcription factor binding site; at, aminotransferase; F, unbound DNA; S, single‐stranded DNA; B, protein‐DNA binding complex; W, well. (a) Electrophoretic mobility shift assays (EMSAs) using a 274 bp fragment covering the intergenic region. The Ah‐BarR protein concentration is indicated in octameric units. In the left panel, the EMSA is performed in the absence of β‐alanine, while in the right panel, a fixed concentration of 5 mM β‐alanine was added. Apparent equilibrium dissociation constants *K*_D_ were determined by Hill curve fitting (Figure A2). (b) EMSAs using operator mutant fragments. (c) In‐gel Cu‐OP footprinting using a 168 bp fragment covering the intergenic region and part of the *ah‐barR* coding region, with the bottom strand being ^32^P‐labeled. A, T, G, and C represent the sequencing ladders, “−” corresponds to input DNA, and “+” corresponds to the Ah‐BarR‐DNA complex. Protected areas are indicated with a green vertical line, while the predicted binding sites are indicated with a gray box, and the TATA box/BRE elements are indicated with light/dark blue boxes, respectively. The protection/hyperreactivity zones are indicated on the DNA sequence. Nucleotides of the bottom strand are colored relative to the degree of protection (values range from 0.3 to 4.3). Predicted binding sites are indicated with a gray box, and the TATA box/BRE element with light/dark blue boxes, respectively. (d) Consensus sequence for Ah‐BarR binding, based on the three identified binding sites TFBS 1, TFBS 2, and TFBS 3.

**Figure A2:**
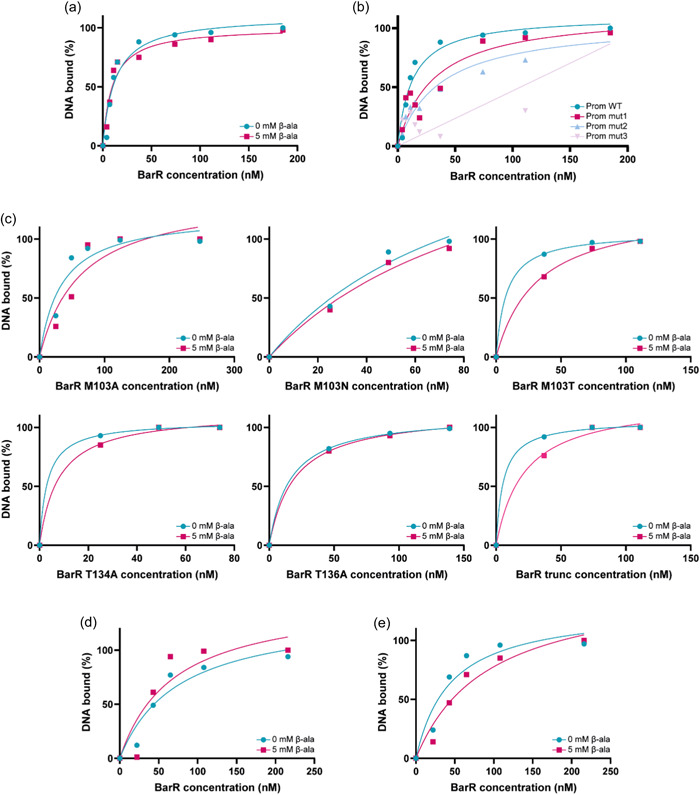
Hill curve fitting enabling quantification of the binding of Ah‐BarR WT or mutants to different DNA fragments. (a) Binding of WT Ah‐BarR to the 274 nt native promoter fragment (corresponding to Figure 2a). (b) Binding of WT Ah‐BarR to the 274 nt mutated promoter fragments (mut1, mut2, and mut3) (corresponding to Figure 2b). (c) Binding of BarR mutant proteins (M103A, M103N, M103T, T134A, T136A, and trunc) to the 274 nt native promoter fragment (corresponding to Figure A6b). (d) Binding of WT Ah‐BarR to the 274 nt native promoter fragment (corresponding to Figure A6a). (e) Binding of WT Ah‐BarR to the 168 nt native promoter fragment (corresponding to Figure A6a).

To unravel the DNA‐binding properties of Ah‐BarR in vitro, EMSA experiments were conducted (Figure 2a,b). The interaction was tested using a 274‐bp DNA fragment harboring the entire intergenic region and the initial open reading frame (ORF) portion of each of the two divergent genes (Table A4). Starting from an octameric Ah‐BarR concentration of 7 nM, DNA‐protein complex formation was observed (Figure 2a). A single complex with low relative mobility was formed, pointing to a high‐MW complex due to the binding of the octameric protein. At Ah‐BarR concentrations of 185 nM and higher, nonspecific interactions and/or protein aggregation prevented protein‐DNA complexes to penetrate the gel. Hill curve fitting confirmed the high‐affinity nature of the interaction, with an apparent equilibrium dissociation constant *K*_D_ of 12 nM (Figure A2). In contrast to Sa‐BarR and St‐BarR, for which it was shown that β‐alanine causes dissociation of protein‐DNA complexes in vitro (Liu et al., 2014), the addition of 5 mM β‐alanine to binding reactions did not significantly alter the formation of Ah‐BarR‐DNA complexes (Figures 2a and A2).

Next, EMSAs were performed using operator mutant fragments to establish the role of each of the predicted binding motifs TFBS1, TFBS2, and TFBS3 for Ah‐BarR binding (Figure 2b). In these operator mutant fragments, the last three bps of one or more predicted binding sites were mutated (CWR to TTT, with W = A/T and R = A/G), either only in TFBS1 (mut1), both in TFBS1 and TFBS2 (mut2) or in all three sites (mut3). Upon mutating TFBS1 (mut1), complex formation was still observed, albeit with a lowered binding affinity (*K*_D_ of 31 nM vs. 12 nM for the WT operator) (Figure 2b and A2). For the mut2 operator mutant fragment, the effect on binding affinity was similar as observed for mut1 (*K*_D_ of 36 nM); however, two complexes were formed instead of one, each with different relative mobility and smearing was observed for the mut2 fragment, pointing to the lower stability of the nucleoprotein complexes causing dissociation during electrophoresis. In the EMSA with the triple mutant (mut3), the binding of Ah‐BarR was completely abolished (Figure 2b). These experiments indicate that all three predicted binding sites contribute to complex formation.

To perform a high‐resolution contact probing of the interaction between Ah‐BarR and the intergenic region, a footprinting experiment was performed (Figure 2c). To this end, a shorter 168‐bp fragment of the intergenic region was subjected to an “in‐gel” copper‐phenanthroline (Cu‐OP) footprinting procedure with the bottom strand labeled, enabling to separate the Ah‐BarR complexes from unbound DNA (input DNA) for a separate analysis. It was observed that both TFBS 2 and TFBS 3 were specifically protected by Ah‐BarR in Ah‐BarR‐DNA complexes, while the promoter of the aminotransferase gene remained unaffected (Figure 2c). Contrarily to TFBS 2 and TFBS 3, TFBS 1 did not seem to be protected by Ah‐BarR, which can be explained by multiple possible reasons: (i) this binding site is further removed from the ^32^P‐labeled end of the labeled strand than the other two binding sites, thereby limiting the resolution of the footprint, making it harder to discern protected areas and ii) based on the binding analysis to operator mutant fragments (Figure 2b), it can be hypothesized that TFBS 1 has less favorable binding kinetics (as compared to TFBS 2 and TFBS 3) that are not as easily captured by chemical footprinting (e.g., due to higher dissociation rate). Not only protection but also hyperreactivity zones were observed in the footprinting experiment, especially in the spacer region between TFBS 2 and TFBS 3 (Figure 2c), pointing to the establishment of protein‐induced DNA deformations.

The consensus sequence for Ah‐BarR binding, based on all three binding site sequences (Figure 2d), displays a high similarity to that of Sa‐BarR (Liu et al., 2014), with an AT‐rich center and palindromic half‐sites. Less favorable binding kinetics for TFBS 1 might be explained by the presence of a C‐T bp on position 10 in the AT‐rich center and/or by an imperfect second‐half site (CTA instead of CAA).

### 3.3 Architectural conformation of Ah‐BarR‐DNA complexes

**Figure 3:**
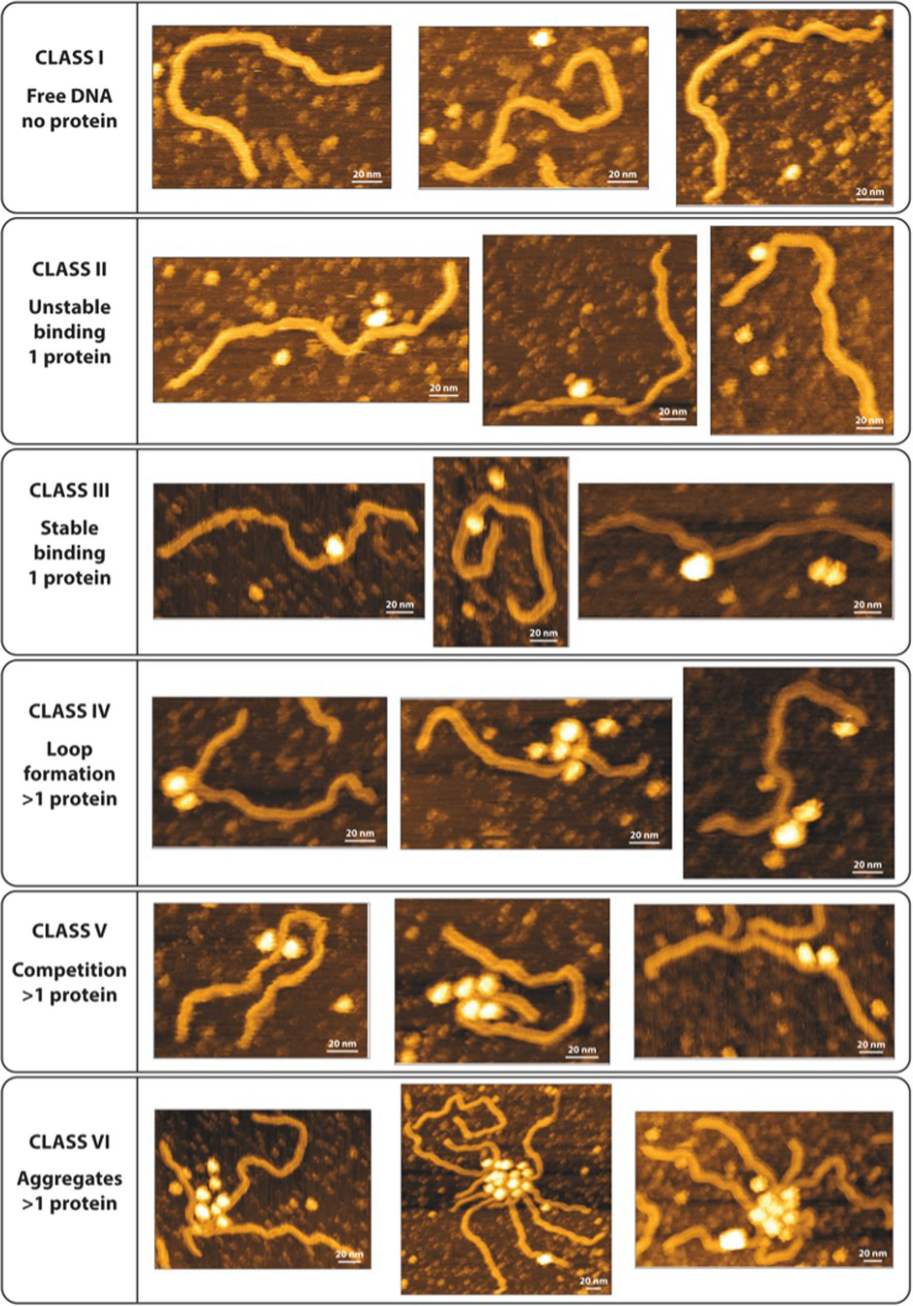
Overview of the different classes of binding species obtained in atomic force microscopy (AFM) with Ah‐BarR and a 780 bp operator fragment. Three topographic images are depicted per class and a 20 nm scale bar is indicated on each image.

AFM, a technique that allows the visualization of DNA and proteins in vitro, was used to study the architectural conformation of the formed Ah‐BarR‐DNA complexes. These experiments enabled us to verify if and how Ah‐BarR deformed DNA, possibly leading to wrapping, as suggested by the footprinting experiments and in previous hypotheses for the *Sulfolobus* homologs (Liu et al., 2014). AFM in liquid was performed on a sample containing a 780‐bp DNA fragment harboring the intergenic region (Table A4) and Ah‐BarR protein (Figure 3).

**Figure A3:**
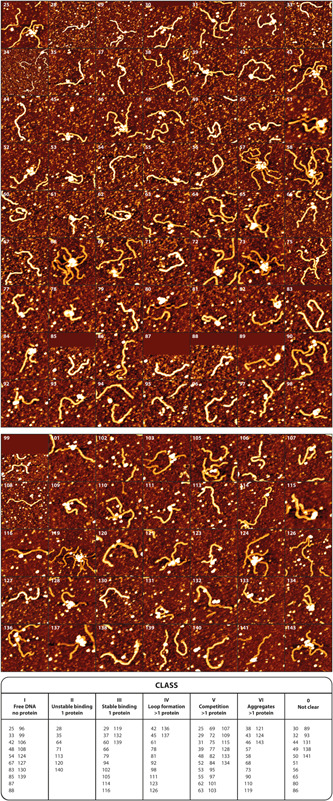
Overview of all atomic force microscopy (AFM) images containing isolated Ah‐BarR‐DNA binding complexes. Image sizes range between 170 × 170 nm and 600 × 600 nm. The table depicts the distribution of images amongst the different classes.

Despite the use of fixed protein and DNA concentrations, heterogenous individual Ah‐BarR‐DNA complexes were visualized in the AFM experiments (Figures 3 and A3). Based on their appearance, six different classes were defined, which represent the different time events of the binding of Ah‐BarR to the DNA. Class I comprises free DNA molecules to which no proteins were visibly bound. In class II and class III, binding of a single large protein oligomer to DNA was observed: while in class II complexes, the Ah‐BarR protein appeared to be only loosely bound, in class III complexes the protein seemed more stably bound with even a wrapping conformation that was observed for the DNA (cfr the third image). Given that Ah‐BarR forms a homogenous population of octamers in solution, it can be hypothesized that these complexes harbor a single octamer that is bound to the specific binding region. Indeed, this binding region is asymmetrically positioned on the fragment, generating complexes with one shorter and one long arm of free DNA.

Complexes with higher stoichiometries were classified in classes IV, V, and VI and might not be prevalent in physiologically relevant conditions. Class IV consisted of structures with clear DNA loop formation, in which one central protein was wrapped by the DNA and extra protein(s) were bound externally to the formed loop. In class V, competition was observed between multiple proteins present in the binding region. The final class, class VI, constituted aggregates in which multiple DNA fragments and proteins were involved. These complexes might correspond to the observed phenomenon of complexes formed at higher protein concentrations that were unable to penetrate the gel in the EMSAs (Figure 2a).

### 3.4 Transcription regulation by Ah‐BarR can be monitored with a heterologous system in a bacterial host

In contrast to *S. acidocaldarius*, *A. hospitalis* is not accessible for genetic experiments. We, therefore, sought an alternative approach to study the mechanisms of transcription regulation and ligand response of Ah‐BarR. To this end, a heterologous Ah‐BarR‐specific reporter gene assay was developed in the model bacterium *E. coli*. Biosensor strains were built by combining an inducible *ah‐barR* expression plasmid (pITC *ah‐barR*), in which *ah‐barR* expression was placed under the control of a naringenin‐inducible promoter, with a reporter plasmid (pPRC). The latter plasmid harbors the intergenic *ah‐barR*‐aminotransferase promoter region in different configurations fused to a reporter gene expressing mKate2 (Table 1). For biosensor strain 1 (BS1), the reporter plasmid pPRC6 P_*barR*_ was used, fusing the *ah‐barR* promoter to the *mkate2* reporter gene, while for biosensor strain 2 (BS2), pPRC6 P_*at*_ was used, containing the promoter of the aminotransferase gene. Additional strains containing an empty pITC plasmid combined with pPRC6 P_*barR*_ or pPRC6 P_*at*_, were referred to as “noTF” and were used as a negative control to anticipate the possible occurrence of leaky expression of the naringenin promoter.

**Figure 4:**
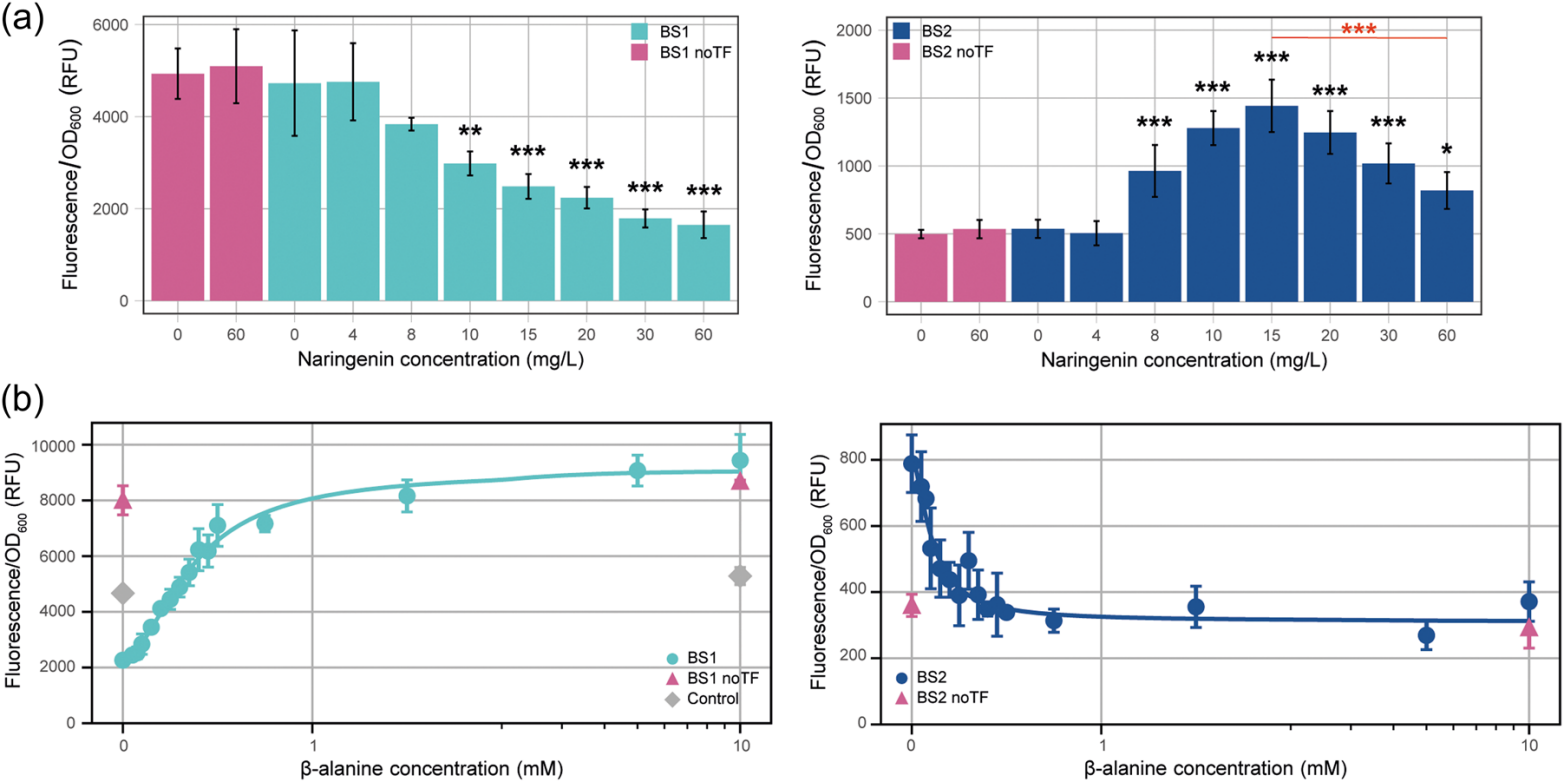
Effect of naringenin‐induced Ah‐BarR expression and addition of different β‐alanine concentrations on mKate2 expression of strains BS1 and BS2, harboring *Acidianus hospitalis* promoters P_*barR*_ and P_*at*_, respectively. Fluorescence/OD_600_ is expressed in relative fluorescence units (RFU). Each point corresponds to the corrected mean relative fluorescence over three time points of four biological replicates, error bars indicate the standard deviation across four replicates. BS1, BS2, BS1/2 noTF, and Control are respectively colored in turquoise, dark blue, pink, and gray. Analysis of statistical significance is provided (Table A5). (a) Response of BS1 and BS2 to different naringenin concentrations (range 0–60 mg/L). *p < 0.05; **p < 0.01; ***p < 0.001 (calculated with one‐way analysis of variance [ANOVA] and Tukey's HSD test) are indicated in comparison to the noTF 0 mg/L naringenin condition. The red asterisks indicate the significant decrease observed for the 60 mg/L condition (compared to the 15 mg/L naringenin condition) of BS2. (b) Response of BS1 and BS2 to different concentrations of β‐alanine at a fixed concentration of 20 mg/L naringenin. Concentrations of β‐alanine range from 0 to 10 mM. Responses of BS1 and BS2 to β‐alanine are displayed on a symmetrical logarithmic *x*‐axis along with their fit to a Hill function (see also Section 2.6).

The biosensor strains were grown in the presence of an increasing naringenin concentration ranging from 0 to 60 mg/L and their fluorescence and OD_600_ were measured (Figure 4a). On the one hand, FL/OD_600_ levels significantly decreased for BS1 (*ah‐barR* promoter) upon Ah‐BarR expression, indicating that Ah‐BarR functions as a transcriptional repressor of its own promoter in this heterologous system. On the other hand, it was observed that for BS2 (*at* promoter), FL/OD_600_ levels increased until a concentration of 15 mg/L naringenin was reached, after which it gradually decreased again, although remaining at higher levels than the negative control (0 mg/L naringenin) (Figure 4a). These observations indicate that Ah‐BarR functions as an activator of the aminotransferase gene and the decrease in FL/OD_600_ might be caused by protein aggregation at higher, physiologically less relevant concentrations. It should be noted that BS1 reached higher fluorescence values as compared to BS2, pointing to a higher promoter strength for P_*ah‐barR*_ than for P_*ah‐at*_ in *E. coli*. These results demonstrate that Ah‐BarR is capable of simultaneously repressing transcription of its own gene and activating transcription of the aminotransferase target gene while being bound as a single octamer to the intergenic region of the divergent operon.

### 3.5 Ah‐BarR is capable of activating and repressing transcription in a β‐alanine responsive manner

**Figure A4:**
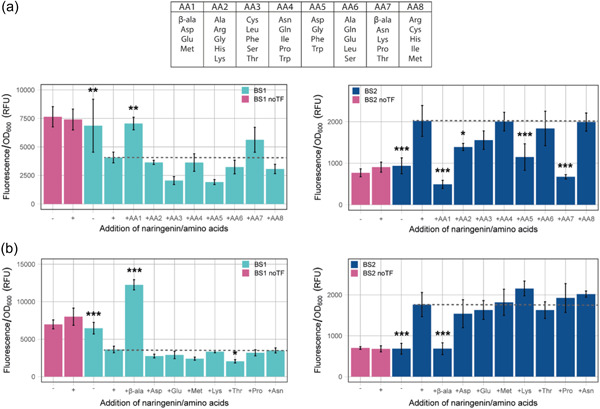
Effect of amino acids on the expression level of BS1 and BS2. Fluorescence/OD_600_ is expressed in relative fluorescence units (RFU). Each point corresponds to the corrected mean relative fluorescence over three timepoints of four biological replicates, error bars indicate the calculated standard deviation over the four replicates. BS1, BS2, BS1/2 noTF, and Control are respectively colored in light blue, dark blue, pink, and gray. A “−” corresponds to a concentration of 0 mg/L naringenin, while a “+” corresponds to a concentration of 20 mg/L naringenin. *p < 0.05; **p < 0.01; ***p < 0.001 (calculated with one‐way analysis of variance [ANOVA] and Tukey's HSD test) are indicated in comparison to the “+” condition (Table A7). (a) Response of BS1 and BS2 to addition of eight different amino acid pools (amino acids added in concentration of 3/5 mM) to the medium. (b) Response of BS1 and BS2 to addition of amino acids from pool 1 and 7 (amino acids added in concentration of 3/5 mM) to the medium.

To characterize ligand specificity, additional experiments were performed with BS1 and BS2 strains in the presence of a fixed naringenin concentration (20 mg/L) and different amino acids, divided into pools of 4–5 individual amino acids (Figure A4a). Amino acid pools 1 and 7 showed the most pronounced effects on transcriptional regulation of *barR* and *at*, after which it was confirmed by testing individual amino acids that this effect could be specifically ascribed to β‐alanine (Figure A4b).

The β‐alanine response was characterized in more detail by measuring dose–response curves for a range of β‐alanine concentrations (from 0 to 10 mM) (Figure 4b). For BS1, it was observed that β‐alanine had a reciprocal effect on the Ah‐BarR‐mediated repression, causing derepression. This response was characterized by a threshold value θ of 0.37 mM and Hill coefficient *n* of 1.77 (Table 2), indicating that a significant response already takes place at low β‐alanine concentrations. For BS2, an opposite effect was noticed, with Ah‐BarR exerting a transcriptional activation in the absence of the ligand and with β‐alanine alleviating this activation to a transcriptional level similar to that of the noTF strain. Although this response was very sensitive to β‐alanine (threshold θ of 0.11 mM), the dynamic range as well as the absolute levels of transcriptional expression, indicated by fluorescence/OD_600_, were lower for BS2 (*at* promoter) as compared to BS1 (*barR* promoter) (Figure 4B, Table 2).

**Figure A5:**
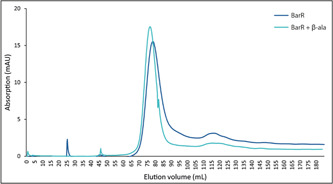
Influence of β‐alanine on oligomeric state of Ah‐BarR. Result of gel filtration experiment of Ah‐BarR and Ah‐BarR supplemented with 100 mM β‐alanine.

**Figure A6:**
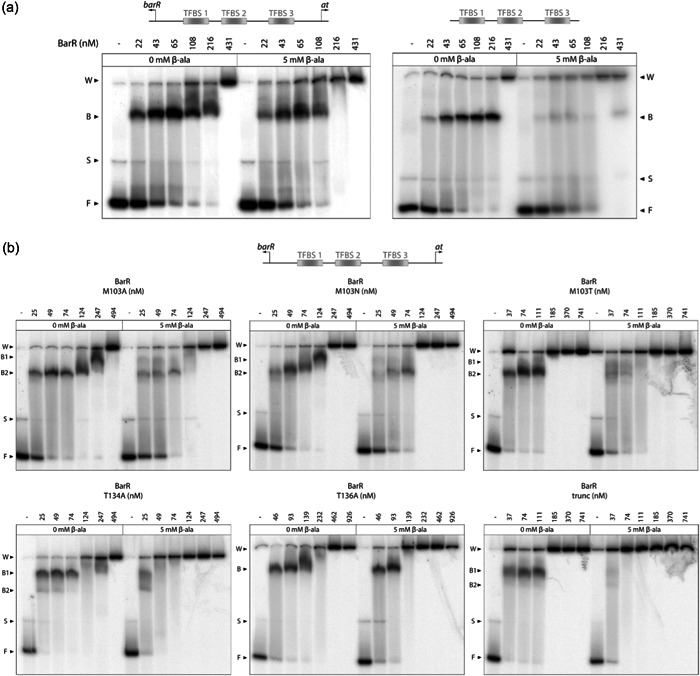
Influence of fragment length and protein mutations on *in vitro* binding of Ah‐BarR to the *ah‐barR*‐aminotransferase intergenic region. BS, binding site; at, aminotransferase; F, unbound DNA; S, single‐stranded DNA; B, protein‐DNA binding complex; W, well. (a) Electrophoretic mobility shift assays (EMSAs) using a 274 bp promoter fragment (left) and a 168 bp promoter fragment (right) and the Ah‐BarR protein at increasing concentrations (octameric concentrations of 0–431 nM), both in absence and presence of 5 mM β‐alanine. (b) EMSAs using a 274 bp promoter fragment and the different Ah‐BarR mutants (M103A, M103N, M103T, T134A, T136A, and trunc) at increasing concentrations (octameric concentrations between 0 and 926 nM), both in absence and presence of 5 mM β‐alanine.

The observation of similar Ah‐BarR‐DNA complex formation behavior in the presence of β‐alanine (Figure 2a), together with the observation of similar SEC elution profiles in the absence and presence of the ligand (Figure A5) leads to the hypothesis that β‐alanine induces small conformational changes in Ah‐BarR while the protein remains bound to DNA and the octameric state of Ah‐BarR remains unaltered. Upon performing an EMSA with a shorter probe of the intergenic region (168 instead of 274 bp), complexes were formed with an unstable behavior during electrophoresis (Figure A6a). This observation corroborates the hypothesis that β‐alanine causes small conformational changes in Ah‐BarR that could alter the behavior of the corresponding Ah‐BarR‐DNA‐complexes, possibly explaining the observed differences in transcriptional output.

**Figure 5:**
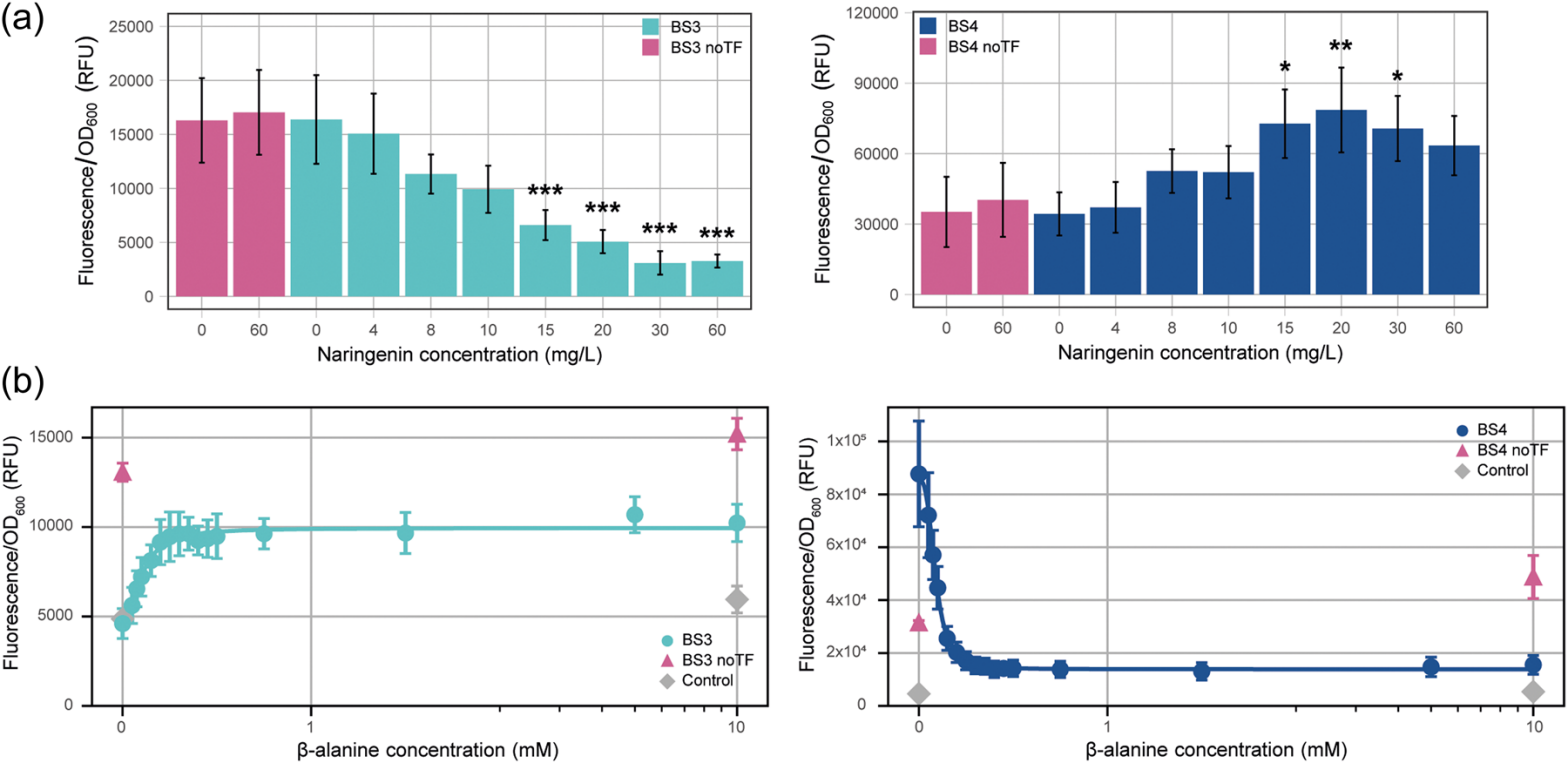
Effect of naringenin‐induced Ah‐BarR expression and addition of different β‐alanine concentrations on the mKate2 expression of strains BS3 and BS4, harboring *Sulfolobus acidocaldarius* promoters P_sa‐barR_ and P_sa‐at_, respectively. Fluorescence/OD_600_ is expressed in relative fluorescence units (RFU). Each point corresponds to the corrected mean relative fluorescence over three time points of four biological replicates, error bars indicate the standard deviation across four replicates. BS3, BS4, BS3/4 noTF, and Control are respectively colored in turquoise, dark blue, pink, and gray. Analysis of statistical significance is provided (Table A5). (a) Response of BS3 and BS4 to different naringenin concentrations (range 0–60 mg/L). *p < 0.05; **p < 0.01; ***p < 0.001 (calculated with one‐way analysis of variance [ANOVA] and Tukey's HSD test) are indicated in comparison to the noTF 0 mg/L naringenin condition. b. Response of BS3 and BS4 to different concentrations of β‐alanine at a fixed concentration of 20 mg/L naringenin. Concentrations of β‐alanine range from 0 to 10 mM. Responses of BS3 and BS4 to β‐alanine are displayed on a symmetrical logarithmic *x*‐axis along with their fit to a Hill function (see also Section 2.6).

Additional in vivo experiments were performed on strains BS3 and BS4 using the *S. acidocaldarius* promoters P_*sa‐barR*_ and P_*sa‐at*_, respectively, in combination with Ah‐BarR. Similar regulatory behavior was observed for BS1 and BS2, although both dose–response curves were characterized by lower threshold values and higher Hill coefficients (Figure 5, Table 2).

### 3.6 Structural determinants of β‐alanine interaction and response

**Figure 6:**
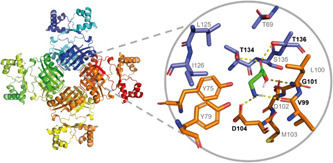
In silico docking of β‐alanine in the ligand binding pocket of Ah‐BarR. Docking was performed in AutoDock Vina for a SWISS‐MODEL‐generated structural model of Ah‐BarR. Residues involved in the formation of the binding pocket are shown. β‐alanine is depicted in green, and chains corresponding to different monomers are colored distinctly (blue vs. orange).

Based on the interactions of the homolog Grp in *S. tokodaii* with its ligand glutamine (Kumarevel et al., 2008), *in silico* docking of β‐alanine was performed for the Ah‐BarR structural model, confirming that this ligand is capable of establishing interactions in a ligand‐binding pocket formed by residues V99, G101, D104, T134, and T136 (Figure 6). These residues correspond to all Grp residues predicted to be involved in ligand interaction with one exception: an asparagine at position 103 in Grp is not conserved and corresponds to threonine in Sa‐BarR and St‐BarR and methionine in Ah‐BarR (Figure 1a).

**Figure 7:**
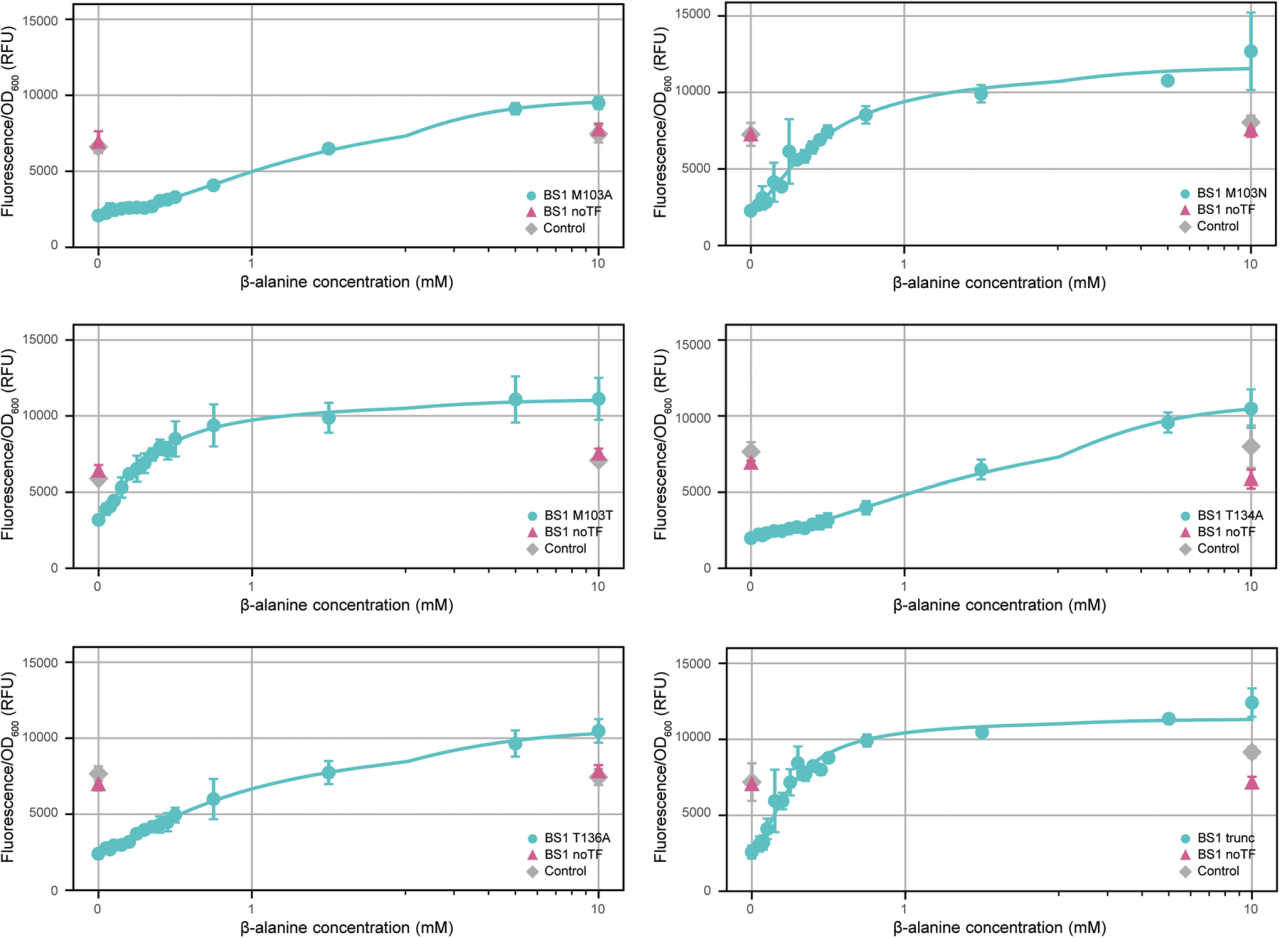
Response of BS1 strains with Ah‐BarR mutants to different concentrations of β‐alanine at a fixed concentration of 20 mg/L naringenin. Concentrations of β‐alanine range from 0 to 10 mM. Fluorescence/OD_600_ is expressed in relative fluorescence units (RFU). Each point corresponds to the corrected mean relative fluorescence over three time points of four biological replicates, error bars indicate the standard deviation across four replicates. Responses of the BS1 mutants (M103A, M103N, M103T, T134A, T136A, and trunc) to β‐alanine are displayed on a symmetrical logarithmic *x*‐axis along with their fit to a Hill function (see also Section 2.6). BS1 mutant, BS1 noTF, and Control are respectively colored in turquoise, pink, and gray. Analysis of statistical significance is provided (Table A6).

The role of residue N103, as well as of T134 and T136, for β‐alanine response in Ah‐BarR was further examined by mutagenesis studies. T134 and T136 are predicted to be of crucial importance for amino acid interaction in all members of the Lrp family (Kawashima et al., 2008). In addition, the importance of the longer C‐terminal tail of Ah‐BarR in comparison with its homologs was also investigated. Six different Ah‐BarR mutants (M103A, M103N, M103T, T134A, T136A, and a truncated mutant) were constructed, confirmed to have an unaltered octameric oligomeric state (Figure 1e), and characterized for DNA binding and transcription regulation, using EMSAs and reporter gene assays, respectively (Figures 7 and A6b).

**Figure A7:**
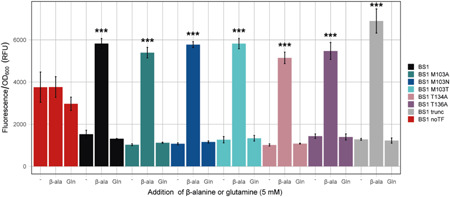
Comparison of β‐alanine and glutamine response of Ah‐BarR WT and mutants. Concentrations of 0 mM (corresponding to “−”) or 5 mM β‐alanine/glutamine are used, together with a fixed concentration of 20 mg/L naringenin. Fluorescence/OD_600_ is expressed in relative fluorescence units (RFU). Each point corresponds to the corrected mean relative fluorescence over three timepoints of four biological replicates. ***p < 0.001 (calculated with one‐way analysis of variance [ANOVA] and Tukey's HSD test) is indicated in comparison to the corresponding ‘−’ condition (Table A8).

Although all mutants were still sensitive to β‐alanine, differences were notable. Reporter gene experiments in *E. coli* showed a significantly decreased sensitivity to β‐alanine for mutants M103A, T134A, and T136A (Figure 7). Although this observation indicates that these residues are indeed important for interaction with β‐alanine, the substitution of M103 for asparagine (M103N) or threonine (M103T), did not significantly alter the β‐alanine response. Moreover, these mutations did not lead to the acquisition of a glutamine‐specific response, indicating that ligand‐binding specificity is determined by other factors (Figure A7). Surprisingly, the truncated mutant showed an increased sensitivity to β‐alanine, with a threshold value of 0.26 mM compared to 0.37 mM for WT Ah‐BarR (Figure 7, Table 2). Also, in terms of DNA‐binding properties, the truncated Ah‐BarR mutant acted differently as compared to WT Ah‐BarR: two distinct complexes were observed in the absence of β‐alanine, while complexes resided in the wells in the presence of β‐alanine (Figure A6).

## 4 DISCUSSION

Our work demonstrates that Ah‐BarR is a dual‐function regulator that is capable of repressing transcription of the promoter of its own gene and of activating transcription of the promoter of a divergently located aminotransferase gene. This was revealed by monitoring the functional regulation of the archaeal regulator and its native promoter/operator region in *E. coli*. Based on these observations, it could be hypothesized that in *A. hospitalis*, Ah‐BarR is capable of simultaneously performing repression and activation of each of the divergently oriented genes while being bound as an octamer to the intergenic region. However, this hypothesis is in contrast to the observation of Sa‐BarR performing auto‐activation in *S. acidocaldarius* (Liu et al., 2014). The observation that in the *E. coli* reporter gene system, transcriptional repression was also observed for the *barR* promoter of *S. acidocaldarius* in combination with Ah‐BarR, indicates that there are differences in regulatory response between the Ah‐BarR system studied in an *E. coli* host and the native Sa‐BarR system (Liu et al., 2014). This might be attributed to host‐specific differences (e.g. the basal transcription machinery), or to differences between Ah‐BarR and Sa‐BarR functionalities, which is unlikely given their high level of sequence identity (Figure 1a). Based on our data, we can therefore not conclude whether or not Ah‐BarR is also a dual‐function regulator in *A. hospitalis*.

**Figure 8:**
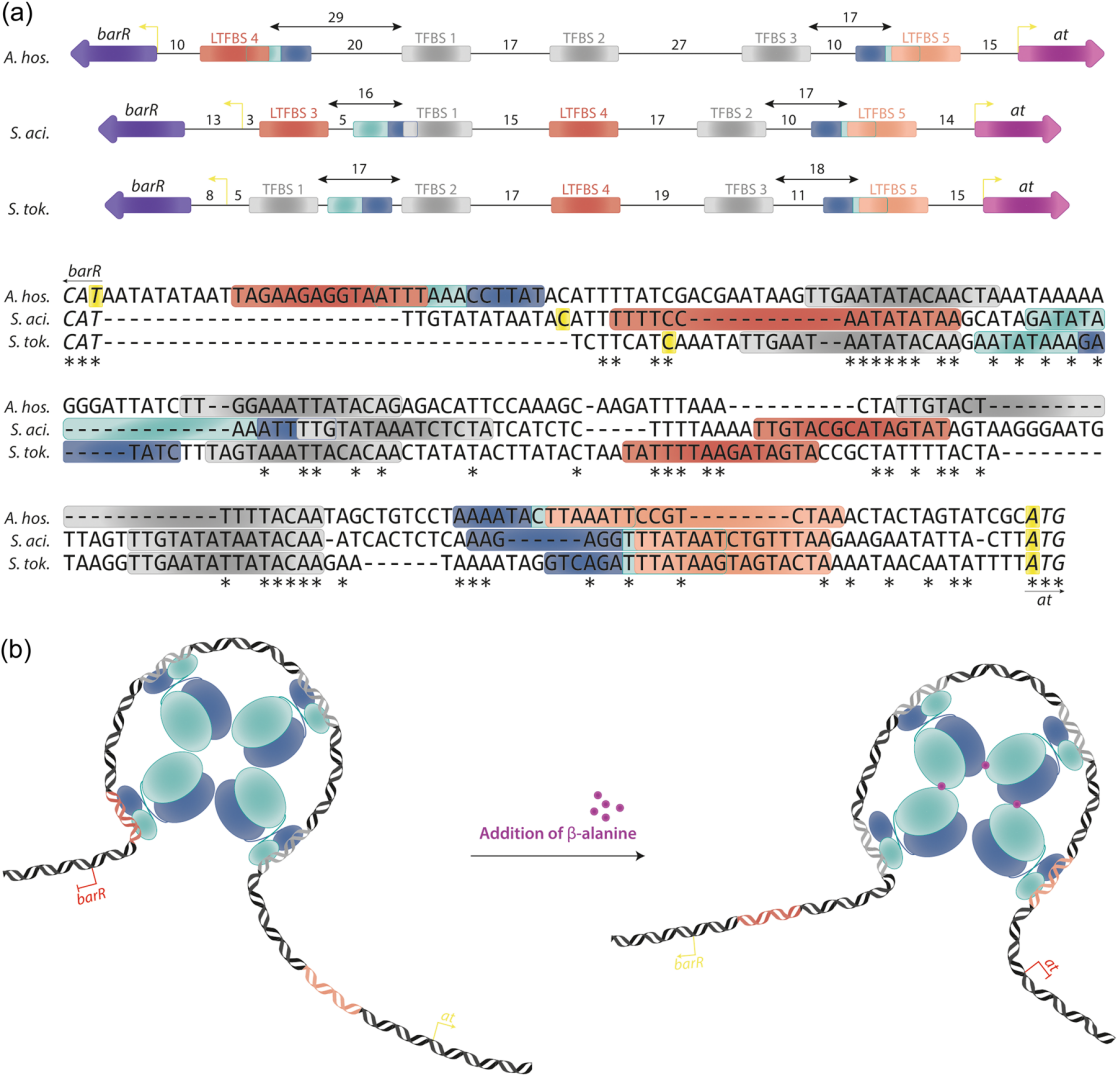
Putative model of DNA binding and interaction with β‐alanine of the transcription factor Ah‐BarR in the order *Sulfolobales*. (a) Schematic overview and multiple sequence alignment of the *barR*‐aminotransferase intergenic region in *Acidianus hospitalis*, *Sulfolobus acidocaldarius*, and *Sulfurisphaera tokodaii*. Confirmed binding sites are indicated with a gray box, predicted low affinity binding sites with a red/orange box, and the TATA box/BRE element, respectively with light/dark blue boxes. Distances (in nucleotides) between the promoter elements are indicated in the figure, and the transcription starts sites are colored yellow. Conserved nucleotides are indicated with an *. (b) Schematic hypothesis of the interaction between *A. hospitalis* BarR, the *barR*‐aminotransferase intergenic region, and β‐alanine. Octameric Ah‐BarR binds binding sites TFBS 1, TFBS 2, TFBS 3, and LTFBS 4 in the absence of β‐alanine, leading to repression of the *barR* gene and activation of the aminotransferase gene. In the presence of β‐alanine, BarR undergoes a conformational change, leading to a change in binding preferences: Ah‐BarR now binds TFBS 1, TFBS 2, TFBS 3, and LTFBS 5, leading to a transcriptional derepression of the *ah‐barR* gene and deactivation of the aminotransferase gene.

It is however clear that the regulatory effect by Ah‐BarR is achieved upon interacting with multiple binding sites in the intergenic region between *barR* and the aminotransferase target gene. Although a similar binding site organization is observed for Ah‐BarR and its homologs Sa‐BarR and St‐BarR, there are small but significant differences (Figure 8a). Sa‐BarR and St‐BarR are predicted to interact with 4 to 5 regularly spaced sites each with spacer lengths between 15 and 17 bp, corresponding to an alignment on the DNA double helix in which similar binding site residues are separated by two helical turns (Liu et al., 2014). The same number of binding sites can be observed or predicted for Ah‐BarR but with differences in spacing. In in vitro experiments, three binding sites were identified for Ah‐BarR in between both promoters and two additional putative auxiliary sites (LTFBS 4 and LTFBS 5) can be predicted based on sequence similarity with the consensus binding motif and/or conservation with respect to the *S. acidocaldarius* and *S. tokodaii* intergenic regions. TFBS 2 and TFBS 3 are separated by 27 bp and LTFBS 4 and TFBS 1 by 29 bp, corresponding to an alignment of three helical turns. Thus, two distinct spacing lengths of 17 bp and 27–29 bp are observed for Ah‐BarR. Notably, the intergenic region between the start codons of both target genes is 27 and 23 bp longer in *A. hospitalis* as compared to *S. acidocaldarius* and *S. tokodaii*, respectively. While the regular binding site spacing of 15–17 bp for Sa‐BarR and St‐BarR corresponds to spacing distances observed for *E. coli* AsnC and *Pyrococcus* sp. OT3 FL11 regulators (Koike et al., 2004; Thaw et al., 2006), the longer spacing of 27‐29 bp also occurs in the binding of *E. coli* Lrp to DNA (de los Rios & Perona, 2007). However, the combination of two different spacing lengths within a single Lrp‐DNA complex has not been observed before. A possible explanation could be that both the differences in spacing of the binding sites and the long C‐terminal tail of BarR have co‐evolved to enable a better adapted regulatory response of the β‐alanine metabolism in *A. hospitalis*. Nevertheless, AFM visualization demonstrated that a complex appears to be formed with the DNA wrapped around an octameric Ah‐BarR molecule (Figure 8b).

Upon interaction with β‐alanine, regulatory effects are relieved while the regulator remains bound to DNA, as is the case for Sa‐BarR (Liu et al., 2016). It is hypothesized that Ah‐BarR undergoes small conformational changes; possibly these changes alter the binding site interaction pattern, for example by releasing the binding site LTFBS4 downstream of the TATA box in P_*barR*_ and binding an additional binding site LTFBS5, which is located at a distance of 17 bp from binding site TFBS3. As a result, interaction with the basal transcription machinery is altered, leading to a derepression of *ah‐bar* transcription and deactivation of *at* transcription. Interesting differences were observed when comparing BarR to its homologs. More specifically, the very long C‐terminal tail and the residue on the position corresponding to M103 were different in all homologs. Mutagenesis studies have indicated that an Ah‐BarR mutant without a tail (*cfr*. truncated mutant) is not only affected by how it interacts with DNA, but it also shows a more sensitive ligand response compared to wild‐type BarR. The functional role of this longer C‐terminal tail remains unclear (Ziegler & Freddolino, 2021), although an allosteric functioning can be proposed. On the other hand, mutational studies have proven that a complete loss of functionality of residue M103 (*cfr*. M103A mutant) leads to a less sensitive β‐alanine response, but that substitution with threonine or asparagine (as observed in Sa‐BarR, St‐BarR, and Grp) restores the sensitivity to the ligand β‐alanine, and not to glutamine as was hypothesized.

## AUTHOR CONTRIBUTIONS

**Amber J. Bernauw**: Formal analysis (lead); funding acquisition (lead); investigation (lead); methodology (lead); visualization (lead); writing—original draft (lead). **Vincent Crabbe**: Formal analysis (supporting); methodology (supporting); visualization (supporting). **Fraukje Ryssegem**: Investigation (supporting); visualization (supporting). **Ronnie Willaert**: Investigation (supporting); methodology (supporting). **Indra Bervoets**: Conceptualization (supporting); writing—review and editing (supporting). **Eveline Peeters**: Conceptualization (lead); funding acquisition (supporting); writing—review and editing (lead).

## CONFLICT OF INTEREST STATEMENT

None declared.

## ETHICS STATEMENT

None required.

## Data Availability

All data are provided in the results and appendix of this paper.

## 1

**Table A1:** List of oligonucleotides used in this work.

Name	Sequence (5′–3′)	Description
AB022	GAAGTGCCATTCCGCCTGAC	FW primer cloning *barR* in pET24a
		FW primer cloning *barR trunc* in pET24a
AB023b	CACTGAGCCTCCACCTAGC	RV primer cloning *barR* in pET24a
AB024	CCCTCAAGACCCGTTTAGAG	FW primer colony PCR pET24a
AB025	ATCTTCCCCATCGGTGATGTC	RV primer colony PCR pET24a
AB100	AAGATCATCTTATTAATCAGATAAAATATTTCTAGACATACTCCTGGAAGTTGGGAAAG	FW primer cloning gb_Ahos in pACYC184
AB101	CGAACGCCAGCAAGACGTAGCCCAGCGCGTCGGCCGCCAGAAGAGATTTTACAGCTCTGG	RV primer cloning gb_Ahos in pACYC184
AB118	GGTAGCTCAGAGAACCTTCGAAAAACC	FW primer colony PCR pACYC184
AB119	CATCTACCTGCCTGGACAGCATG	RV primer colony PCR pACYC184
		RV primer generation probe AFM
AB126	CTTTAAGTCTACCTCGTCTAGCTCC	FW primer generation promoter sequences for EMSA
AB127	GAACTTTCCAAGTTCCGAAAGTATGTTC	RV primer generation promoter sequences for EMSA
AB142	GACGATGGTAGTCAGCTGCCTACTAAGCTGTCTAGAGTAAAAATATACTCCTTCGGCTTTATC	FW primer cloning P _*ah‐barR*_ in pPRC6
AB144	GACGATGGTAGTCAGCTGCCTACTAAGCTGTCTAGAGGCTACTGTTGATTTAGGAACTTTTAC	FW primer cloning P _*ah‐at*_ in pPRC6
AB212	CACACCCCGCCAGGCAGGGTAGGAGACAAGGAGACAGCCATGGAATTGGATGAAGTGGACCTTAAG	FW primer cloning *ah‐barR* in pITC
		FW primer cloning *ah‐barR trunc* in pITC
AB213	GGTTGACAGTTGTTAGCCGATTACGACTCGAGACGATGTCGACTCACTTCCCGTTCTTGATTTCCTCGATG	RV primer cloning *ah‐barR* in pITC
AB256	CATGTTTTCTTTGATCAGCTCGCTAACCATAATATATAATTAGAAGAGGTAATTTAAACC	RV primer cloning P _*ah‐barR*_ in pPRC6
AB257	CATGTTTTCTTTGATCAGCTCGCTAACCATGCGATACTAGTAGTTTAGACG	RV primer cloning P _*ah‐at*_ in pPRC6
AB338	GACGATGGTAGTCAGCTGCCTACTAAGCTGTCTAGAGTCCAGGTACTTATTACCTTTGG	FW primer cloning P_*sa‐barR*_ in pPRC6
AB339	CATGTTTTCTTTGATCAGCTCGCTAACCATTTGTATATAATACATTTTTTCCAATATATAAGC	RV primer cloning P_*sa‐barR*_ in pPRC6
AB340	GACGATGGTAGTCAGCTGCCTACTAAGCTGTCTAGACGAGCCTCTTAATCCTATATGC	FW primer cloning P_*sa‐at*_ in pPRC6
AB341	CATGTTTTCTTTGATCAGCTCGCTAACCATAAGTAATATTCTTCTTAAACAGATTATAAACC	RV primer cloning P_*sa‐at*_ in pPRC6
AB354	CCTTATACATTTTATCGACGAATAAGTTG	FW primer generation promoter sequences for EMSA and footprinting
AB386	TGTACTTCGTGCTGGGCGACGCGGATTTCGTGGTTCTGGCCCG	FW primer site directed mutagenesis *ah‐barR* M103A
AB387	CGGGCCAGAACCACGAAATCCGCGTCGCCCAGCACGAAGTACA	RV primer site directed mutagenesis *ah‐barR* M103A
AB388	TGTACTTCGTGCTGGGCGACAACGATTTCGTGGTTCTGGCCCG	FW primer site directed mutagenesis *ah‐barR* M103N
AB389	CGGGCCAGAACCACGAAATCGTTGTCGCCCAGCACGAAGTACA	RV primer site directed mutagenesis *ah‐barR* M103N
AB390	TGTACTTCGTGCTGGGCGACACCGATTTCGTGGTTCTGGCCCG	FW primer site directed mutagenesis *ah‐barR* M103T
AB391	CGGGCCAGAACCACGAAATCGGTGTCGCCCAGCACGAAGTACA	RV primer site directed mutagenesis *ah‐barR* M103T
AB392	ACATGCCGGAGATCGAGCGTGCGTCGACGCATGTGGTGGCGAA	FW primer site directed mutagenesis *ah‐barR* T134A
AB393	TTCGCCACCACATGCGTCGACGCACGCTCGATCTCCGGCATGT	RV primer site directed mutagenesis *ah‐barR* T134A
AB394	CGGAGATCGAGCGTACCTCGGCGCATGTGGTGGCGAAGGTATA	FW primer site directed mutagenesis *ah‐barR* T136A
AB395	TATACCTTCGCCACCACATGCGCCGAGGTACGCTCGATCTCCG	RV primer site directed mutagenesis *ah‐barR* T136A
AB396	CATCCATGCGATACTAGTAGTTTAGACG	RV primer generation promoter sequences for EMSA and footprinting
AB397	CAGCCGGATCTCAGTGGTGGTGGTGGTGGTGCTCGAGGTCAATGGTAACGTAGG	RV primer cloning *ah‐barR* trunc in pET24a
AB398	CCGATTACGACTCGAGACGATGTCGACTCAGTCAATGGTAACGTAGGGGGTCTC	RV primer cloning *ah‐barR* trunc in pITC
AB399	GCTGGGTCAACGTAAGTAAAATAGC	FW primer generation probe AFM
AB400	GAATAAGTTGAATATACAATTTAATAAAAAGGGATTATCTTG	FW primer site directed mutagenesis pACYC184 gb_Ahos mut1
AB401	CAAGATAATCCCTTTTTATTAAATTGTATATTCAACTTATTC	RV primer site directed mutagenesis pACYC184 gb_Ahos mut1
AB402	GATTATCTTGGAAATTATATTTAGACATTCCAAAGCAAG	FW primer site directed mutagenesis pACYC184 gb_Ahos mut2
AB403	CTTGCTTTGGAATGTCTAAATATAATTTCCAAGATAATC	RV primer site directed mutagenesis pACYC184 gb_Ahos mut2
AB404	CTATTGTACTTTTTATTTTAGCTGTCCTAAAATAC	FW primer site directed mutagenesis pACYC184 gb_Ahos mut3
AB405	GTATTTTAGGACAGCTAAAATAAAAAGTACAATAG	RV primer site directed mutagenesis pACYC184 gb_Ahos mut3
IB313	GTGGTTATTCACGGTGCCTTCC	RV primer colony PCR pPRC6
IB442	TGAGCCTGGCCATGACAACG	FW primer colony PCR pITC
IB559	TGACCACTTCGGATTATCCCGTGAC	RV primer colony PCR pITC
IB560	GTCCAGATAGCCCAGTAGCTGACATTC	FW primer colony PCR pPRC6

**Table A2:** Overview of the FastDigest restriction enzymes used for cloning purposes.

Plasmid	Restriction Enzyme 1	Restriction Enzyme 2
pACYC184	Eco52I (EagI)	XbaI
pET24a	NdeI	XhoI
pITC	NcoI	SalI
pPRC6	NdeI	XbaI

**Table A3:** Overview of all plasmid constructs used in this work.

Name	Usage	Reference/Source
pACYC184	Backbone plasmid used for cloning of gb_Ahos	Chang and Cohen (1978)
pACYC184 gb_Ahos	Long term storage of gb_Ahos, template for generation of promoter fragments	This work
pACYC184 gb_Ahos mut1	Template for generation of promoter mut 1 fragment	This work
pACYC184 gb_Ahos mut2	Template for generation of promoter mut 2 fragment	This work
pACYC184 gb_Ahos mut3	Template for generation of promoter mut 3 fragment	This work
pET24a	Backbone plasmid used for cloning of *ah‐barR* WT and mutant genes, negative control in overexpression experiments	Novagen
pET24a *ah‐barR*	Heterologous overexpression and HisTrap purification of Ah‐BarR	This work
pET24a *ah‐barR* M103A	Heterologous overexpression and HisTrap purification of Ah‐BarR M103A	This work
pET24a *ah‐barR* M103N	Heterologous overexpression and HisTrap purification of Ah‐BarR M103N	This work
pET24a *ah‐barR* M103T	Heterologous overexpression and HisTrap purification of Ah‐BarR M103T	This work
pET24a *ah‐barR* T134A	Heterologous overexpression and HisTrap purification of Ah‐BarR T134A	This work
pET24a *ah‐barR* T136A	Heterologous overexpression and HisTrap purification of Ah‐BarR T136A	This work
pET24a *ah‐barR* trunc	Heterologous overexpression and HisTrap purification of Ah‐BarR trunc	This work
pITC	Backbone plasmid used for cloning of *barR* WT and mutant genes, negative control in plate reader experiments	Bernauw et al. (2022)
pITC *ah‐barR*	Construct exhibiting naringenin inducible expression of Ah‐BarR, used in plate reader experiments	This work
pITC *ah‐barR* M103A	Construct exhibiting naringenin inducible expression of Ah‐BarR M103A, used in plate reader experiments	This work
pITC *ah‐barR* M103N	Construct exhibiting naringenin inducible expression of Ah‐BarR M103N, used in plate reader experiments	This work
pITC *ah‐barR* M103T	Construct exhibiting naringenin inducible expression of Ah‐BarR M103T, used in plate reader experiments	This work
pITC *ah‐barR* T134A	Construct exhibiting naringenin inducible expression of Ah‐BarR T134A, used in plate reader experiments	This work
pITC *ah‐barR* T136A	Construct exhibiting naringenin inducible expression of Ah‐BarR T136A, used in plate reader experiments	This work
pITC *ah‐barR* trunc	Construct exhibiting naringenin inducible expression of Ah‐BarR trunc, used in plate reader experiments	This work
pPRC6	Backbone plasmid used for cloning of promoter sequences, negative control in plate reader experiments	This work
pPRC6 P _*ah‐barR*_	Reporter construct containing *mkate2* red fluorescent gene under control of the *barR* gene promoter P _*ah‐barR*_	This work
pPRC6 P _*ah‐at*_	Reporter construct containing *mkate2* red fluorescent gene under control of the aminotransferase gene promoter P _*ah‐at*_	This work
pPRC6 P_*sa‐barR*_	Reporter construct containing *mkate2* red fluorescent gene under control of the *sa‐barR* gene promoter P_*sa‐barR*_	This work
pPRC6 P_*sa‐at*_	Reporter construct containing *mkate2* red fluorescent gene under control of the *S. acidocaldarius* aminotransferase gene promoter P_*sa‐at*_	This work

**Table A4:** Overview of fragments created for EMSA, footprinting and AFM.

Name	Template	Used primers	Length (bp)	Usage
WT promoter	pACYC184 gb_Ahos	AB126 & AB127	274	EMSA
Promoter mutant 1	pACYC184 gb_Ahos mut1	AB126 & AB127	274	EMSA
Promoter mutant 2	pACYC184 gb_Ahos mut2	AB126 & AB127	274	EMSA
Promoter mutant 3	pACYC184 gb_Ahos mut3	AB126 & AB127	274	EMSA
WT promoter short	pACYC184 gb_Ahos	AB354 & AB396	168	EMSA, footprinting
WT promoter long	pACYC184 gb_Ahos	AB399 & AB119	780	AFM

**Table A5:** Statistical significance assessment performed to compare FL/OD_600_ values obtained for biosensors BS1, BS2, BS3, and BS4 grown at different naringenin or β‐alanine concentrations.

**Experiment: naringenin gradient General score (all conditions)**	**BS1 (Figure** 4a)	**BS2 (Figure** 4b)	**BS3 (Figure** 5a **)**	**BS4 (Figure** 5b **)**
	***p*‐value ANOVA**	***p*‐value ANOVA**	***p*‐value ANOVA**	***p*‐value ANOVA**
	4.14E‐11	1.36E‐12	1.61E‐09	5.16E‐05
**Per condition**	***p*‐value Tukey**	***p*‐value Tukey**	***p*‐value Tukey**	***p*‐value Tukey**
0 Nar (noTF)—60 Nar (noTF)	0.9999931	0.9999941	0.9999955	0.9999067
0 Nar (noTF)—0 Nar	0.9999519	0.9999905	1	1
0 Nar (noTF)—4 Nar	0.9999868	1	0.9996838	1
0 Nar (noTF)—8 Nar	0.2186077	0.0008364	0.2710374	0.7018981
0 Nar (noTF)—10 Nar	0.0014341	0.0000001	0.0621499	0.7312985
0 Nar (noTF)—15 Nar	0.0000500	0.0000000	0.0007649	0.0122569
0 Nar (noTF)—20 Nar	0.0000097	0.0000002	0.0000866	0.0024444
0 Nar (noTF)—30 Nar	0.0000005	0.0001668	0.0000053	0.0212191
0 Nar (noTF)—60 Nar	0.0000002	0.0457724	0.0000067	0.1229396
15 Nar—60 Nar	/	0.0000079	/	/

**Experiment: β‐alanine gradient** **General score (all conditions)**	**BS1 (Figure** 4c **)**	**BS2 (Figure** 4d **)**	**BS3 (Figure** 5c **)**	**BS4 (Figure** 5d **)**
	***p*‐value ANOVA**	***p*‐value ANOVA**	***p*‐value ANOVA**	***p*‐value ANOVA**
	<2E‐16	<2E‐16	<2E‐16	<2E‐16
**Per condition**	***p*‐value Tukey**	***p*‐value Tukey**	***p*‐value Tukey**	***p*‐value Tukey**
0 β‐ala (noTF)—10 β‐ala (noTF)	0.938756	0.9958423	0.5276236	0.3366217
0 β‐ala (noTF)—0 β‐ala	0.000000	0.0000000	0.0000000	0.0000000
0 β‐ala—0.05 β‐ala	1	0.9943629	0.9943100	0.2769996
0 β‐ala—0.075 β‐ala	0.9999995	0.7986326	0.4353740	0.0000699
0 β‐ala—0.1 β‐ala	0.9861096	0.0005539	0.0657507	0.0000000
0 β‐ala—0.15 β‐ala	0.2260662	0.0000071	0.0016524	0.0000000
0 β‐ala—0.2 β‐ala	0.0030927	0.0000006	0.0000125	0.0000000
0 β‐ala—0.25 β‐ala	0.0002526	0.0000000	0.0000027	0.0000000
0 β‐ala—0.3 β‐ala	0.0000086	0.0000399	0.0000013	0.0000000
0 β‐ala—0.35 β‐ala	0.0000002	0.0000000	0.0000012	0.0000000
0 β‐ala—0.4 β‐ala	0.0000000	0.0000000	0.0000080	0.0000000
0 β‐ala—0.45 β‐ala	0.0000000	0.0000000	0.0000048	0.0000000
0 β‐ala—0.5 β‐ala	0.0000000	0.0000000	0.0000025	0.0000000
0 β‐ala—0.75 β‐ala	0.0000000	0.0000000	0.0000012	0.0000000
0 β‐ala—1.5 β‐ala	0.0000000	0.0000000	0.0000010	0.0000000
0 β‐ala—5 β‐ala	0.0000000	0.0000000	0.0000000	0.0000000
0 β‐ala—10 β‐ala	0.0000000	0.0000000	0.0000001	0.0000000

*Note*: A one‐way analysis of variance (ANOVA) and Tukey's HSD test were performed for each experiment, leading to the given *p*‐values. For the Tukey's HSD test, only comparisons of the relevant conditions are shown in this table. Nonsignificant values (*p* > 0.05) are colored in red.

**Table A6:** Statistical significance assessment performed to compare FL/OD_600_ values obtained for the BS1 mutants grown at different β‐alanine concentrations.

**Experiment: β‐alanine gradient mutants (Figure** 7 **)** **General score (all conditions)**	**BS1 M103A**	**BS1 M103N**	**BS1 M103T**	**BS1 T134A**	**BS1 T136A**	**BS1 trunc**
	***p*‐value ANOVA**	***p*‐value ANOVA**	***p*‐value ANOVA**	***p*‐value ANOVA**	***p*‐value ANOVA**	***p*‐value ANOVA**
	<2E‐16	<2E‐16	2.54E‐16	<2E‐16	<2E‐16	<2E‐16
**Per condition**	***p*‐value Tukey**	***p*‐value Tukey**	***p*‐value Tukey**	***p*‐value Tukey**	***p*‐value Tukey**	***p*‐value Tukey**
0 β‐ala (noTF)—10 β‐ala (noTF)	0.0991541	1	0.8261439	0.1541542	0.754957	1
0 β‐ala (noTF)—0 β‐ala	0.0000000	0.000001	0.0004700	0.0000000	0.000000	0.0000001
0 β‐ala—0.05 β‐ala	1	1	0.9994928	0.9999982	0.999924	0.9999633
0 β‐ala—0.075 β‐ala	0.9884033	0.9990308	0.9931645	1	0.999999	0.9995708
0 β‐ala—0.1 β‐ala	0.9969570	0.9999958	0.8579750	0.9998965	0.991916	0.4405306
0 β‐ala—0.15 β‐ala	0.9654030	0.4812945	0.1263954	0.9928421	0.986785	0.0001043
0 β‐ala—0.2 β‐ala	0.9138457	0.7528035	0.0033468	0.9926892	0.846376	0.0001089
0 β‐ala—0.25 β‐ala	0.8843080	0.0004801	0.0006832	0.9010324	0.094984	0.0000002
0 β‐ala—0.3 β‐ala	0.9233317	0.0046425	0.0001208	0.7464743	0.016614	0.0000000
0 β‐ala—0.35 β‐ala	0.7442931	0.0018100	0.0000075	0.8687800	0.003158	0.0000000
0 β‐ala—0.4 β‐ala	0.0808606	0.0001603	0.0000008	0.3852859	0.000915	0.0000000
0 β‐ala—0.45 β‐ala	0.0418474	0.0000191	0.0000023	0.1850560	0.000224	0.0000000
0 β‐ala—0.5 β‐ala	0.0086033	0.0000019	0.0000001	0.0593087	0.000003	0.0000000
0 β‐ala—0.75 β‐ala	0.0000017	0.0000000	0.0000000	0.0000194	0.000000	0.0000000
0 β‐ala—1.5 β‐ala	0.0000000	0.0000000	0.0000000	0.0000000	0.000000	0.0000000
0 β‐ala—5 β‐ala	0.0000000	0.0000000	0.0000000	0.0000000	0.000000	0.0000000
0 β‐ala—10 β‐ala	0.0000000	0.0000000	0.0000000	0.0000000	0.000000	0.0000000

*Note*: A one‐way analysis of variance (ANOVA) and Tukey's HSD test were performed for each experiment, leading to the given *p*‐values. For the Tukey's HSD test, only comparisons of the relevant conditions are shown in this table. Nonsignificant values (*p* > 0.05) are colored in red.

**Table A7:** Statistical significance assessment performed to compare FL/OD_600_ values obtained for BS1 and BS2 biosensors tested with AA pools and addition of separate amino acids.

**Experiment: AA pools** **General score (all conditions)**	**BS1 (Figure** A4a **)**	**BS2 (Figure** A4a **)**
	***p*‐value ANOVA**	***p*‐value ANOVA**
	1.45E‐12	3.04E‐13
**Per condition**	***p*‐value Tukey**	***p*‐value Tukey**
0 Nar (noTF)—20 Nar (noTF)	0.9999998	0.9993721
0 Nar—20 Nar	0.0060270	0.0000064
20 Nar—20 Nar+AA1	0.0025997	0.0000000
20 Nar—20 Nar+AA2	0.9999125	0.0213412
20 Nar—20 Nar+AA3	0.1145407	0.2104216
20 Nar—20 Nar+AA4	0.9998909	1
20 Nar—20 Nar+AA5	0.0722273	0.0003218
20 Nar—20 Nar+AA6	0.9740418	0.9928239
20 Nar—20 Nar+AA7	0.4431204	0.0000001
20 Nar—20 Nar+AA8	0.9128982	1

**Experiment: separate amino acids** **General score (all conditions)**	**BS1 (Figure** A4b **)**	**BS2 (Figure** A4b **)**
	***p*‐value ANOVA**	***p*‐value ANOVA**
	<2E‐16	6.28E‐14
**Per condition**	***p*‐value Tukey**	***p*‐value Tukey**
0 Nar (noTF)—20 Nar (noTF)	0.3003688	1
0 Nar—20 Nar	0.0000007	0.0000041
20 Nar—20 Nar+β‐ala	0.0000000	0.0000042
20 Nar—20 Nar+Asp	0.5306215	0.9567567
20 Nar—20 Nar+Glu	0.7677627	0.9993145
20 Nar—20 Nar+Met	0.1113371	0.9999999
20 Nar—20 Nar+Lys	0.9997801	0.3848392
20 Nar—20 Nar+Thr	0.0119674	0.9992448
20 Nar—20 Nar+Pro	0.9916769	0.9960937
20 Nar—20 Nar+Asn	1	0.8954723

*Note*: A one‐way analysis of variance (ANOVA) and Tukey's HSD test were performed for each experiment, leading to the given *p*‐values. For the Tukey's HSD test, only comparisons of the relevant conditions are shown in this table. Nonsignificant values (*p* > 0.05) are colored in red.

**Table A8:** Statistical significance assessment performed to compare FL/OD_600_ values obtained for the BS1 mutants grown in the presence of 0 or 5 mM β‐alanine/glutamine.

Experiment: β‐ala and gln response (Figure A7)	BS1 noTF	BS1 WT	BS1 M103A	BS1 M103N	BS1 M103T	BS1 T134A	BS1 T136A	BS1 trunc
General score (all conditions)	*p*‐value ANOVA	*p*‐value ANOVA	*p*‐value ANOVA	*p*‐value ANOVA	*p*‐value ANOVA	*p*‐value ANOVA	*p*‐value ANOVA	*p*‐value ANOVA
	0.194	1.09E‐07	4.36E‐08	1.44E‐09	1.16E‐07	9.71E‐08	1.57E‐06	1.21E‐06
**Per condition**	***p*‐value Tukey**	***p*‐value Tukey**	***p*‐value Tukey**	***p*‐value Tukey**	***p*‐value Tukey**	***p*‐value Tukey**	***p*‐value Tukey**	***p*‐value Tukey**
0–5 mM β‐ala	0.9997681	0.0000004	0.0000001	0.0000000	0.0000003	0.0000003	0.0000027	0.0000021
0–5 mM gln	0.2476110	0.3475487	0.7283329	0.5080209	0.8847865	0.8857757	0.9792420	0.9760398

*Note*: A one‐way analysis of variance (ANOVA) and Tukey's HSD test were performed for each experiment, leading to the given *p*‐values. For the Tukey's HSD test, only comparisons of the relevant conditions are shown in this table. Nonsignificant values (*p* > 0.05) are colored in red.
